# Systematic analysis of the role of LDHs subtype in pan-cancer demonstrates the importance of LDHD in the prognosis of hepatocellular carcinoma patients

**DOI:** 10.1186/s12885-024-11920-8

**Published:** 2024-01-31

**Authors:** Shengnan Wang, Xingwei Wu, Xiaoming Wu, Jin Cheng, Qianyi Chen, Zhilin Qi

**Affiliations:** 1 Department of Biochemistry and Molecular Biology, Wannan Medical College, No.22 Wenchang West Road, Wuhu, Anhui 241002 P.R. China; 2 Anhui Province Key Laboratory of Active Biological Macro-Molecules, Wannan Medical College, Wuhu, Anhui 241002 P.R. China; 3 Department of Pathology, Fuyang People’s Hospital, Anhui Medical University, Fuyang, Anhui 236000 P.R. China; 4 Clinical Laboratory, Traditional Chinese Hospital of Lu’an, Anhui University of Chinese Medicine, Lu’an 237000, Anhui, P.R. China; 5 Department of Thyroid and Breast Surgery, Yijishan Hospital, First Affiliated Hospital of Wannan Medical College, Wuhu, Anhui 241002 P.R. China; 6 Department of Gastroenterology, Yijishan Hospital, First Affiliated Hospital of Wannan Medical College, Wuhu, Anhui 241002 P.R. China

**Keywords:** LDHD, HCC, Bioinformatics, Prognostic biomarkers, Pan-cancer

## Abstract

**Background:**

Lactate dehydrogenase (LDHs) is an enzyme involved in anaerobic glycolysis, including LDHA, LDHB, LDHC and LDHD. Given the regulatory role in the biological progression of certain tumors, we analyzed the role of LDHs in pan-cancers.

**Methods:**

Cox regression, Kaplan–Meier curves, Receiver Operating Characteristic (ROC) curves, and correlation of clinical indicators in tumor patients were used to assess the prognostic significance of LDHs in pan-cancer. The TCGA, HPA, TIMER, UALCAN, TISIDB, and Cellminer databases were used to investigate the correlation between the expression of LDHs and immune subtypes, immune checkpoint genes, methylation levels, tumor mutational load, microsatellite instability, tumor-infiltrating immune cells and drug sensitivity. The cBioPortal database was also used to identify genomic abnormalities of LDHs in pan-cancer. A comprehensive assessment of the biological functions of LDHs was performed using GSEA. In vitro*,* HepG2 and Huh7 cells were transfected with LDHD siRNA and GFP-LDHD, the proliferation capacity of cells was examined using CCK-8, EdU, and colony formation assays; the migration and invasion of cells was detected by wound healing and transwell assays; western blotting was used to detect the levels of MMP-2, MMP-9, E-cadherin, N-cadherin and Akt phosphorylation.

**Results:**

LDHs were differentially expressed in a variety of human tumor tissues. LDHs subtypes can act as pro-oncogenes or anti-oncogenes in different types of cancer and have an impact on the prognosis of patients with tumors by influencing their clinicopathological characteristics. LDHs were differentially expressed in tumor immune subtypes and molecular subtypes. In addition, LDHs expression correlated with immune checkpoint genes, tumor mutational load, and microsatellite instability. LDHD was identified to play an important role in the prognosis of HCC patients, according to a comprehensive analysis of LDHs in pan-cancer. In HepG2 and Huh7 cells, knockdown of LDHD promoted cell proliferation, migration, and invasion, promoted the protein expression levels of MMP-2, MMP-9, N-cadherin, and Akt phosphorylation, but inhibited the protein expression level of E-cadherin. In addition, LDHD overexpression showed the opposite changes.

**Conclusion:**

LDHs subtypes can be used as potential prognostic markers for certain cancers. Prognostic and immunotherapeutic analysis indicated that LDHD plays an important role in the prognosis of HCC patients. In vitro experiments revealed that LDHD can affect HCC proliferation, migration, and invasion by regulating MMPs expression and EMT via Akt signaling pathway, which provides a new perspective on the anti-cancer molecular mechanism of LDHD in HCC.

**Supplementary Information:**

The online version contains supplementary material available at 10.1186/s12885-024-11920-8.

## Introduction

Cancer remains one of the greatest public health challenges today, with a significant impact on human health and social development due to a steady increase in incidence and mortality rates [1]. Global cancer statistics of 2020 show an estimated 19.3 million newly diagnosed cancer cases and nearly 10 million deaths worldwide in 2020 only [2]. The most common cancers are breast, lung, colorectal, prostate, and stomach [3–5]. Exposure to different carcinogenic factors, normal cells lose their regulatory growth mechanism at the genetic level, resulting in uncontrolled cell proliferation, unlimited growth, and highly invasive and metastatic behavior. With the advent of molecularly targeted and immunotherapies in recent years, a significant decrease in cancer-induced deaths has been observed [6, 7]. However, due to emerging resistance to immunotherapy, there is an urgent need to develop reliable diagnostic and prognostic biomarkers for cancer.

Cancer is a highly heterogeneous disease whose development requires different processes of tissue cell metabolism, where metabolic remodeling can influence the biological function of tumors [8]. The correlation between aerobic glycolysis and cancer is the biochemical basis for the development of novel anti-cancer strategies, where LDHs are of pivotal importance among different enzymes involved in glycolysis [9, 10]. Most tumor cells inhibit mitochondria oxidative phosphorylation and instead increase glucose consumption and lactate, which is consumed independently of oxygen production (Warburg effect) [11]. Therefore, energy production in cancer cells is abnormally dependent on glycolysis, and targeting aerobic glycolysis may be helpful for therapeutic interventions in cancer [12].

LDH, a tetrameric enzyme, is an important metabolic enzyme whose inhibition could block aerobic glycolysis in tumor cells [13]. The family of LDH enzymes includes LDHA, LDHB, LDHC, and LDHD. LDHA and LDHB are elevated in many tumor types and are associated with tumor growth and invasion [14]. LDHA has been reported to regulate HCC tumor growth and metastasis by inducing MMP-2 production, which leads to apoptosis by inhibiting the production of reactive oxygen species (ROS) [15]. In addition, it may be a key enzyme in the conversion of pyruvate to lactate, which is highly expressed in BRCA, and inhibiting its expression may provide a new therapeutic strategy for the treatment of BRCA [16]. LDHA, in combination with mTORC1 or MAPK inhibitors has been shown to affect the progression of SKCM [17]. Similarly, reduced expression of LDHB in BRCA may confer a growth and survival advantage over BRCA [18]. Low expression of LDHB can promote PAAD progression by inducing a glycolytic phenotype [19]. HYOU1 has been reported to encourage tolerance of glucose and malignant progression in thyroid cancer cells by upregulating LDHB expression [20]. On the other hand, LDHC has been found to promote PI3K/Akt/GSK-3β process in LUAD cells and has been shown to play an essential role in BRCA migration and invasion [21, 22]. Furthermore, the downregulation of LDHD expression may be an important prognostic indicator for patients with clear ccRCC, and overexpression of LDHD might contribute to UCEC development [23, 24]. Several studies indicate that LDHs may play a key role in the biological progression of certain cancers. However, these studies have only focused on a limited number of tumor types and the function of LDHs in pan-cancer has not yet been investigated.

DNA methylation is an important epigenetic modification in mammals, DNA methylation silences a wide range of genes, therefore aberrant methylation modifications translate into abnormal gene expression, which may play a vital role in cancer development [25]. Studies have shown that tumor mutation burden (TMB) is associated with immunotherapy response and can predict immune checkpoint inhibitors (ICI) response [26]. Microsatellite instability (MSI) is a hypermutated phenotype that results in loss of DNA mismatch repair (MMR) activity. MSI occurs at different frequencies in malignancies and can predict cancer response/resistance to certain chemotherapies [27]. Additionally, there is a close relationship between the tumor microenvironment (TME) and the efficacy of immunotherapy, where tumor-infiltrating cells in the TME can influence the immune profile of malignant tumors [28]. Immune checkpoint (ICP) inhibitors have potent tumor suppressive effects, and the study of gene-ICP correlations is essential to inhibit malignant tumor proliferation.

Different tumors can inhibit their clearance and lysis by the immune system through various pathways, leading to immune tolerance. Inter-individual tumor heterogeneity may affect the efficacy of clinical immunotherapy [29]. Therefore, there is an urgent need to develop personalized treatment plans to mitigate the damage caused by overtreatment. Precision medicine has yet to be fully expressed in cancer treatment and requires urgent attention to explore better therapeutic targets [30].

LDHs subtype has the potential to serve as cancer diagnostic markers. This study systematically analyzed LDHs subtype from the aspects of mRNA expression, methylation, mutation patterns, immune infiltration, functional enrichment analysis, clinically relevant prognosis and potential chemotherapeutic agents in pan-cancer. The results suggested that LDHD plays an important role in the clinical prognosis and immunotherapy treatment of HCC patients. Therefore, we will focus on the role of LDHD in HCC patients and the underlying molecular mechanisms involved.

## Materials and methods

### TCGA database

RNA-seq (FPKM) gene expression data for different cancer types were downloaded from the open-access database UCSC-Xena. The gene expression profile data were log2-transformed for comparisons between groups to make gene expression data more comparable between samples. LDHs expression levels in the downloaded data identified 33 different cancer types, where expression differences between tumor and normal tissue samples were identified by a p-value < 0.05 criterion. For the TCGA pan-cancer analysis, four LDHs gene expression levels were provided, and differences between para-cancerous and tumor tissue samples were assessed using Student's t-test, excluding cancer types with low numbers of normal samples. Internal correlations of LDHs were examined using “pheatmap" and "corrplot" design in R. Differential expression of LDHs between tumor types in the TCGA database and corresponding normal tissues was analyzed using the TIMER database. The correlation of LDHs expression in patients' clinical information with different cancer types was analyzed using R software, which was combined, followed by using R software "ggpubr" package for statistical analysis to visualize the patients' pathological, histological, T, M and N stage, and to screen the clinical indicators with significant differences.

### Analysis of the relationship between LDHs and prognosis

Survival data were downloaded from the TCGA database for samples from different cancer types, and overall survival (OS), progression-free interval (PFI), disease-free interval (DFI), and disease-free survival (DSS) were considered indicators to explore the correlation between LDHs and patient prognosis. The median expression of LDHs was used as a threshold to classify high and low-expression subgroups, and the Kaplan–Meier method and log-rank sum test were used for each cancer type. Survival" and "Survminer" were used to plot survival curves. The R package "forestplot" was used to analyze the relationship between the LDHs expression and pan-cancer survival. To assess the diagnostic accuracy of LDHs in patients with different types of cancer, ROC curves based on sensitivity and specificity were performed using the "pROC" package. In addition, Cox analysis was performed to determine the correlation between LDHs and disease prognosis, and finally, the R package "forestplot" was used to plot graphs and perform univariate Cox regression analysis on LDHs expression.

### Immunohistochemical staining (IHC)

IHC images of LDHs protein expression in normal tissues and corresponding tumor tissues of different cancer types were obtained from the HPA database (http://www.proteinatlas.org), and three cancer types with significant differences, including thyroid, lung, and kidney cancer were selected to explore the differences in protein expression.

### Biological functions of LDHs based on GSEA (gene enrichment analysis)

Kyoto Encyclopedia of Genes and Genes (KEGG) analysis was performed using GSEA in an online database (http://www.gsea-msigdb.org/). The biological functions of LDHs in different cancer types were analyzed by GSEA. The study was conducted using the R packages "limma,” "clusterprofiler,” "org,Hs,eg,db" and The "enrichplot" package was used for visualization.

### Genomically altered LDHs in pan-cancer

The cBioportal database (http://www.cbioportal.org) contains all oncogene data from the TCGA database and can be used to analyze pan-cancer data. Data from 10,953 samples from 32 cancer types were selected to analyze the types and frequencies of mutations in LDHs genes in all tumors. To analyze the mutations of LDHs in the TCGA pan-cancer dataset, the "Oncoprint,” "CancerType Summary," and "Mutations" modules were used to obtain information on genetic alterations and mutation loci of LDHs. In addition, "CancerType Summary" showed the mutation rate of LDHs genes in pan-cancer as a bar graph.

### Correlation of LDHs expression with DNA methylation

UALCAN (http://ualcan.path.uab.edu/), an analysis database based on TCGA gene data, was used in this study to analyze the methylation levels of LDHs in different cancer types.

### Relationship between the expression of LDHs and immune cells

The relative scores of immune cells in different cancer types were calculated using CIBERSORT, which can predict different immune cell phenotypes. The packages "ggplot2", "ggpubr," and "ggExtra" based on R software were used to analyze the correlation between LDHs and the level of infiltration of each immune cell.

### Correlation between LDHs expression and molecular subtypes and immunomodulators in different cancer types

TISIDB (cis.hku.hk/TISIDB) is a database for analyzing tumor gene expression and immune system interactions. The correlation between LDHs expression levels, molecular subtypes, and immunomodulators (Immuno-inhibitor, Immuno-stimulator, and MHC molecule) in different cancer types was investigated using the TISIDB database. Immune Subtype correlations between LDHs and BLCA, BRCA, KIRC, KIRP, LGG, LIHC, LUAD, OV, PRAD, and UCEC were analysed using the R packages "limma", "ggplot2", and "reshape2".

### Correlation analysis of LDHs expression with immune checkpoints

Correlation analysis between LDHs expression levels and different immune checkpoint genes (ICP) was analyzed using Spearman correlation analysis. The "ESTIMATE" and "limma" packages of the R package were used to calculate stromal and immune cell scores in different cancer types to assess the level of LDHs expression in the stromal and immune cell score infiltration.

### Correlation analysis of LDHs expression with TMB and MSI

Pearson correlation coefficients between LDHs expression and TMB, MSI, DNAss, and RNAss in different cancer types were analyzed using the R packages "limma" and "corrplot.” TMB and MSI were calculated using TCGA cell mutation data, and a radar plot was created to analyze the relationship between LDHs and TMB and MSI using Pearson's correlation analysis.

### Drug sensitivity analysis

NCI-60 chemical activity data and the corresponding RNA-seq expression dataset were downloaded from CellMiner (http://discover.nci.nih.gov/cellminer/home.do) to analyze the drug sensitivity of LDHs in pan-cancer, followed by limma", and "ggplot,” and the results were visualized using the "limma,” "ggplot2″ and "ggpubr" packages in R software to explore the potential correlation between LDHs expression and drug sensitivity.

### Antibodies & reagents

The EdU cell proliferation detection kit assay was purchased from RiboBio Co., Ltd. (Guangzhou, China). Cell Counting Kit-8 (CCK-8) was purchased from KeyGen Biotech Co., Ltd (Nanjing, China). LDHD protein primary antibody (K008489P) was purchased from Solarbio (Beijing, China). Anti-GAPDH (D16H11) and anti-p-Akt (Ser473) antibodies were purchased from Cell Signaling Technology (Beverly, MA, USA). The antibodies against E-cadherin (A20798), N-cadherin (A19083), MMP-9 (A0289), and MMP-2 (A6247) were the products of ABclonal Biotechnology (Wuhan, China). Horseradish peroxidase-linked anti-mouse IgG and anti-rabbit IgG secondary antibodies were purchased from Cell Signaling Technology (Beverly, MA, USA).

### Cell culture and transfection

The human hepatoma cell line HepG2 and Huh7 was purchased from Fuheng Cell Center (Shanghai, China), which was authenticated by STR profiling. HepG2 and Huh7 cell lines were cultured in MEM and DMEM medium supplemented with 10% FBS, respectively. The siRNA targeting LDHD (LDHD siRNA) and negative control siRNA (si-NC) were designed and synthesized by RiboBio. LDHD siRNA (100 nM) and si-NC were transfected into cells using the riboFECT™ CP transfection kit (RiboBio, Guangzhou, China) according to the manufacturer's protocols. LDHD overexpression (GFP-LDHD) and negative plasmids were purchased from GeneChem Co. (Shanghai, China), HepG2 and Huh7 cells were cultured respectively in 6-well plates until 80% confluence, transient transfection was performed using Lipofectamine 3000 reagent (Thermo Fisher Scientific, Inc.) according to the manufacturer's instructions, and transfection efficiency was detected using western blotting.

### CCK-8 assay

The viability of HepG2 and Huh7 cells was determined using the CCK-8 assay. Briefly, HepG2 and Huh7 cells transfected with LDHD siRNA or GFP-LDHD plasmid and negative control were seeded into 96-well cell culture plates, respectively. Following incubation for 24 h, 48 h, or 72 h, 10 µl/well of CCK-8 working fluid was added. After incubation for another 2 h, the optical density (OD) values of each well were measured at 450 nm using a Multiskan™ GO plate reader (Thermo Fisher Scientific, Inc.). The experiment was repeated three times and data were expressed as mean ± SD.

### EdU assay

HepG2 and Huh7 cells transfected with LDHD siRNA or GFP-LDHD and negative control were seeded into 24-well plates and cultured at 37℃ and 5% CO_2_ for 24 h, their proliferation capacity was determined by performing the EdU assay according to the instructions provided by manufacturer. Briefly, after staining with 50 µM of EdU dye, the cells were fixed with 4% paraformaldehyde for 30 min at room temperature and imaged using inverted fluorescence microscopy (Olympus, Tokyo, Japan). The ratio of EdU-positive cells to cells with Hoechst staining was calculated. Image J version 1.52 software was used to analyse the results.

### Colony formation assay

The HepG2 and Huh7 cells transfected with LDHD siRNA or GFP-LDHD and negative control were planted into 6-well plates (500 cells/well) and cultured for 2 weeks. The cells were fixed with 4% paraformaldehyde, stained with 0.1% crystal violet for 30 min at room temperature. After washing for 3 times with PBS, the colonies were counted by Imaging J version 1.52 software.

### Wound healing assay

HepG2 and Huh7 cells transfected with LDHD siRNA or GFP-LDHD and negative control were seeded into 12-well plates and cultured to 90%-100% monolayer confluence. The monolayer-fused cells were scraped gently with a clean 200 μl pipette tip, and the detached cells were then removed by washing with PBS. The distance of cell migration was observed by light microscopy (Olympus) at 0 h, 24 h and 48 h respectively. The results were analysed using ImageJ version 1.52 software.

### Transwell assay

HepG2 and Huh7 cells transfected with LDHD siRNA or GFP-LDHD and negative control were respectively resuspended with 200 µl FBS-free MEM or DMEM medium and then plated in the upper chamber, and 600 µl of medium containing 20% FBS was added to the lower chamber. Similarly, CIM Plate 16 upper chambers precoated with diluted Matrigel (356234; BD Biosciences) were used for Matrigel invasion assay. After 24 h of incubation in an incubator at 37℃ and 5% CO_2_, the cells on the upper surface were removed with cotton swabs gently, and the migrated cells were fixed with 4% paraformaldehyde for 30 min and stained with 0.1% crystal violet for 20 min. After washing with PBS, the images of migrated cells were captured using an inverted fluorescence microscope (Olympus).

### Protein extraction and Western blot

HepG2 and Huh7 cells were seeded in 6-well plates, and then transfected with LDHD siRNA or GFP-LDHD and negative control for the indicated times. After washing with cold PBS, the cells were lysed with RIPA cell lysis buffer containing protease inhibitors (Beyotime, Haimen, China). The lysates were centrifuged (12000 g) at 4℃ for 10–15 min. The amounts of total protein were quantified by NanoDrop one (Thermo Fisher). For Western blotting, equivalent amounts of protein (50 µg) were loaded and separated using 12% or 10% SDS-PAGE and then the proteins were transferred onto nitrocellulose membranes (Pall Corporation, Port Washington, NY, USA). The membranes were blocked with 5% skimmed milk for 1 h at room temperature, washed with TBST for three times and probed with the indicated primary antibodies overnight at 4℃. The membrane was incubated with HRP-conjugated secondary antibodies for 2 h at room temperature following three washes with TBST. The antigen–antibody complexes were detected using a chemiluminescence imaging system (Clinx, Shanghai, China).

### Statistical analysis

Statistical significance between the two groups was analysed using student’s t-test, while the Wilcoxon rank sum test and Spearman's rank test were used to compare the differences in the expression of LDHs and the correlation between tumor and normal tissues, respectively. All R package analysis were performed using R (version 4.2.1), except for the online website tools. The prognostic role of LDH expression in each cancer type was assessed using a one-way Cox regression analysis, where *p* < 0.05 was considered statistically significant (*p < 0.05, **p < 0.01, ***p < 0.001).

## Results

### Correlation analysis of LDHs-series genes in pan-cancer

**Fig. 1:**
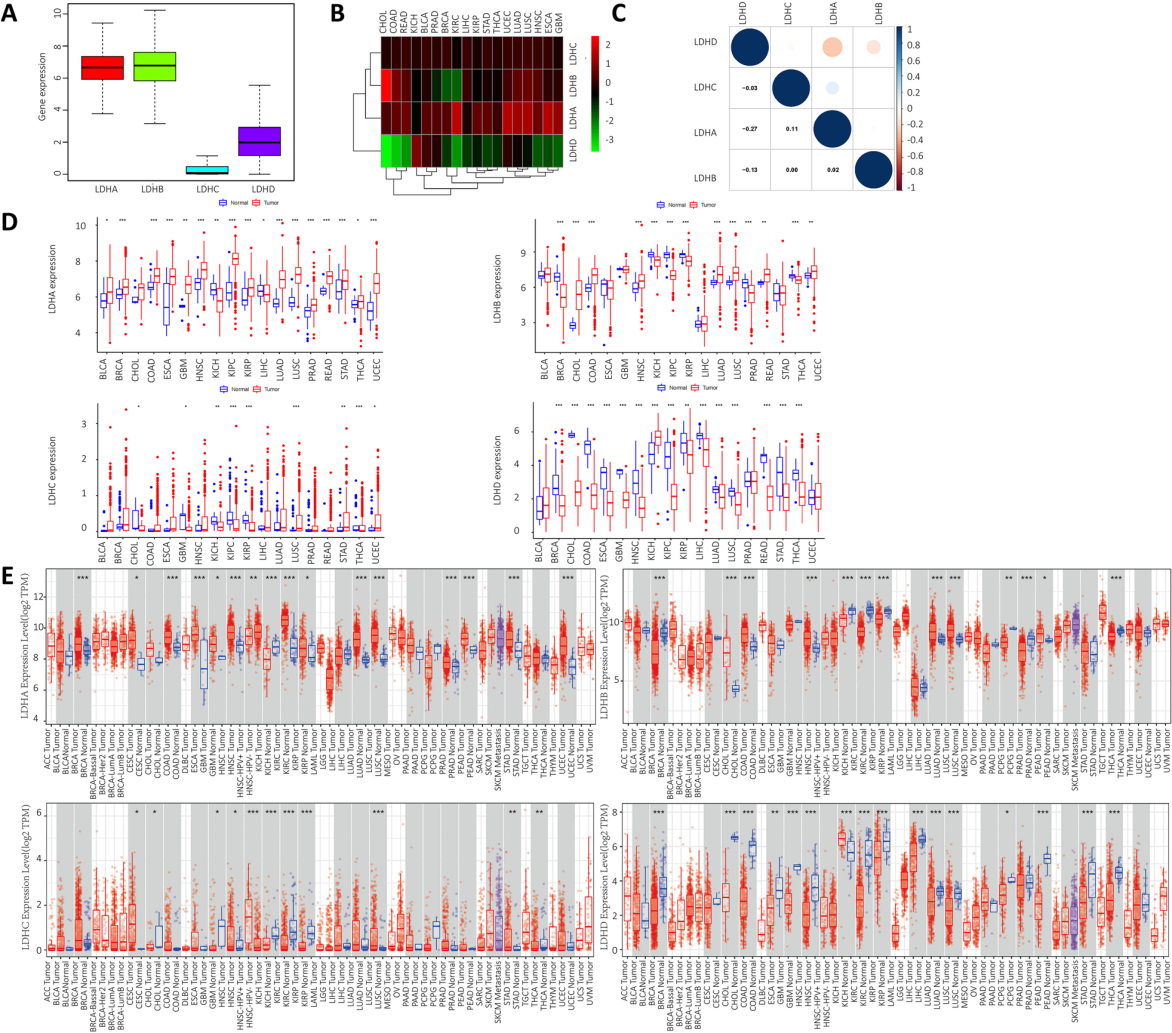
Expression levels and correlations of LDHs family genes in different cancer from the TCGA database. **A** Differential expression levels of LDHs family genes in different types of cancer; **B** Expression data from TCGA database showing expression of LDHs family genes in different cancers; color of each small matrix represents differential expression of LDHs family genes in different cancers, red and green represent high and low expression;** C** Correlation between LDHs family genes, red indicates negative positive correlation and blue indicates positive negative correlation;** D** The expression differences of LDHs family genes in different cancer tissues and normal tissues based on the UCSC-Xena database; **E** The difference expression of LDHs family genes in different cancer tissues and normal tissues from the TIMER database

Analyzing the expression of LDHs genes including LDHA, LDHB, LDHC and LDHD in all cancer types, we found that the expression of LDHA, LDHB and LDHD was higher than that of LDHC in pan-cancer (Fig. 1A). We further analyzed the expression of LDHs genes in 33 cancer types and found that LDHD expression was significantly lower in pan-cancer, especially in CHOL and COAD (Fig. 1B). Analyzing the correlation of LDHs genes expression, we found that the expression of LDHA and LDHC showed the most significant positive correlation, while LDHA and LDHD showed the most significant negative correlation (Fig. 1C). We also extracted the expression of LDHs from the UCSC-Xena database using R software. To avoid statistical error, some cancers were excluded because the number of normal samples was less than 5. Figure 1D showed the expression differences of LDHs in all cancer tissues and paracancerous tissues. In addition, we validated the expression differences of LDHs in different cancer types from the TIMER database (Fig. 1E). The correlation between LDHs expression and prognostic data was then analyzed. Kaplan–Meier survival curves for LDHs are shown in Fig. S1A-D. The results showed that in patients with ACC, CESC, LGG, LIHC, LUAD and PAAD, the survival time of patients with high LDHA expression was shorter than that of patients with low LDHA expression. In patients with HNSC, LIHC, LUAD and SKCM, the survival time of patients with high LDHB expression was shorter than that of patients with low LDHB expression, and in patients with GBM and LGG, the survival time of patients with low LDHB expression was shorter than that of patients with high LDHB expression. In UCEC patients, the survival time of patients with high LDHC expression was shorter than that of patients with low LDHC expression, and in UVM patients, the survival time of patients with low LDHC expression was shorter than that of patients with high LDHC expression. In ACC, CESC, KIRC, KIRP, LUAD and UVM patients, the survival time of patients with low LDHD expression was shorter than that of patients with high LDHD expression. We further examined the IHC results in the HPA database to assess the expression of LDHs in terms of protein levels. Using the HPA database, we analyzed the expression of LDHs in lung, thyroid and kidney tumor tissues. As shown in Fig. S2A-D, LDHA was highly expressed in lung, kidney and thyroid cancer tissues. LDHB was highly expressed in lung cancer tissues but low in kidney and thyroid cancer tissues. LDHC expression was low in kidney and thyroid cancer tissues and has little variability in lung cancer tissues. LDHD expression was low in lung, kidney and thyroid cancer tissues.

### Prognostic significance of LDHs in pan-cancer

**Fig. 2:**
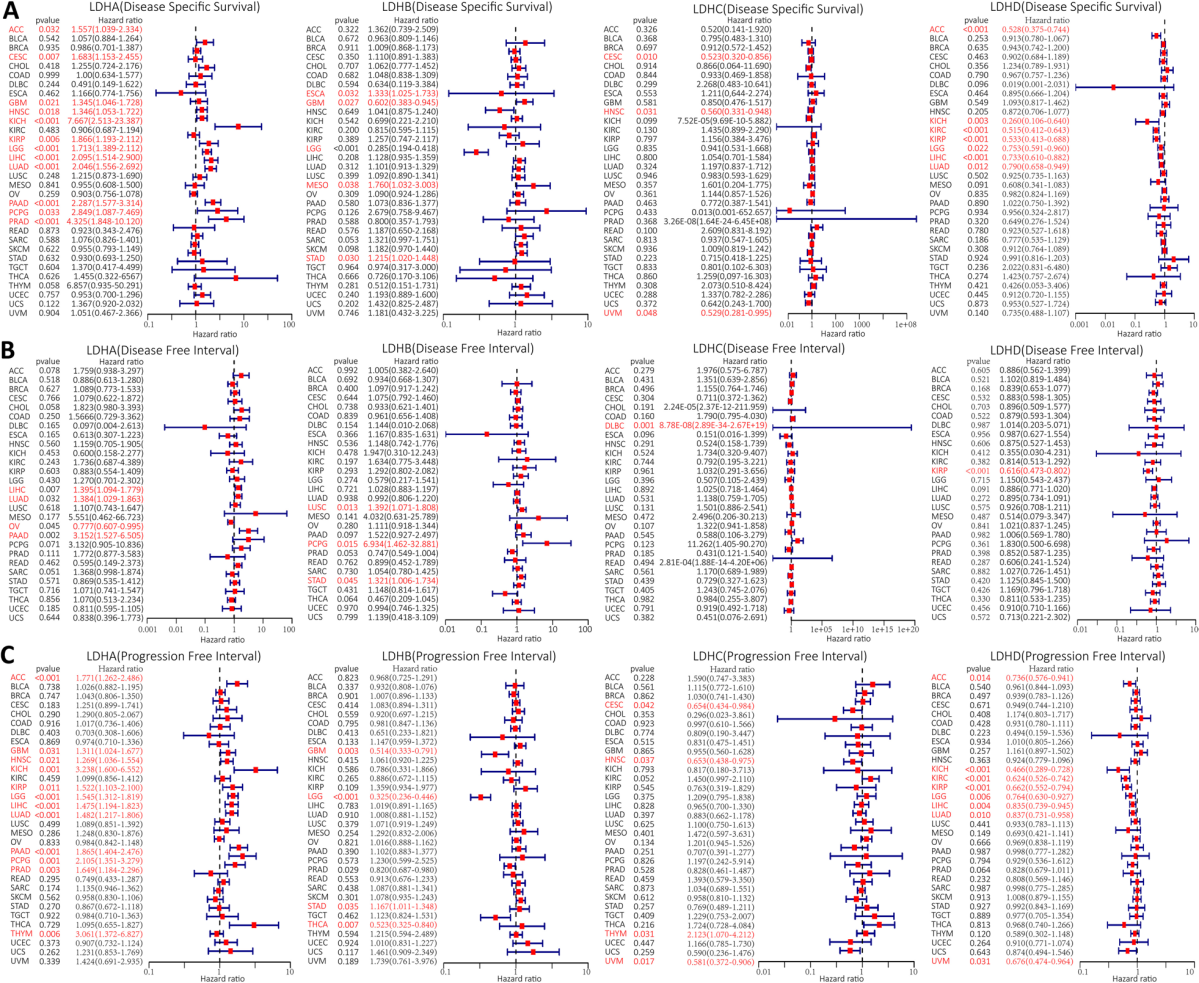
Associations between LDHs expression levels and disease specific survival (DSS), disease free interval (DFI) and progression free interval (PFI).** A** Forest plot of LDHs family gene expression levels in pan-cancer in association with DSS; **B** Forest plot of LDHs family gene expression levels in pan-cancer associated with DFI; **C** Forest plot of LDHs family gene expression levels in pan-cancer associated with PFI

To explore the relationship between LDHs expression levels and prognosis, we divided tumors into high and low expression groups based on the median expression of LDHs in different cancer types, and then investigated the prognostic value of LDHs in the TCGA pan-cancer database by cox regression analysis. The results showed that LDHs subtype had an impact on prognosis in some cancer patients, which were presented as forest plots. As shown in Fig. 2A, the Disease Specific Survival (DSS) data indicated that LDHA expression can significantly affect ACC (HR = 1.557), CESC (HR = 1.683), GBM (HR = 1.345), HNSC (HR = 1.346), KICH (HR = 7.667), KIRP (HR = 1.866), LGG (HR = 1.713) and LIHC (HR = 2.095), LUAD (HR = 2.046), PAAD (HR = 2.287), PCPG (HR = 2.849) and PRAD (HR = 4.325). LDHB expression significantly affected ESCA (HR = 1.333), GBM (HR = 0.602), LGG (HR = 0.285), MESO (HR = 1.760) and STAD (HR = 1.215). The expression of LDHC had a significant effect on CESC (HR = 0.523), HNSC (HR = 0.560) and UVM (HR = 0.529). LDHD expression significantly affected ACC (HR = 0.528), KICH (HR = 0.260), KIRC (HR = 0.515), KIRP (HR = 0.533), LGG (HR = 0.753), LIHC (HR = 0.733) and LUAD (HR = 0.790).

Disease Free Interval (DFI) data analysis showed that LDHA expression could significantly affect LIHC (HR = 1.395), LUAD (HR = 1.384), OV (HR = 0.777) and PAAD (HR = 3.152) (Fig. 2B). LUSC (HR = 1.392), PCPG (HR = 6.934) and STAD (HR = 1.321) were significantly affected by LDHB expression. LDHC expression had a significant effect on DLBC (HR = 8.78E-08). LDHD expression significantly affected KIRP (HR = 0.616).

**Fig. 3:**
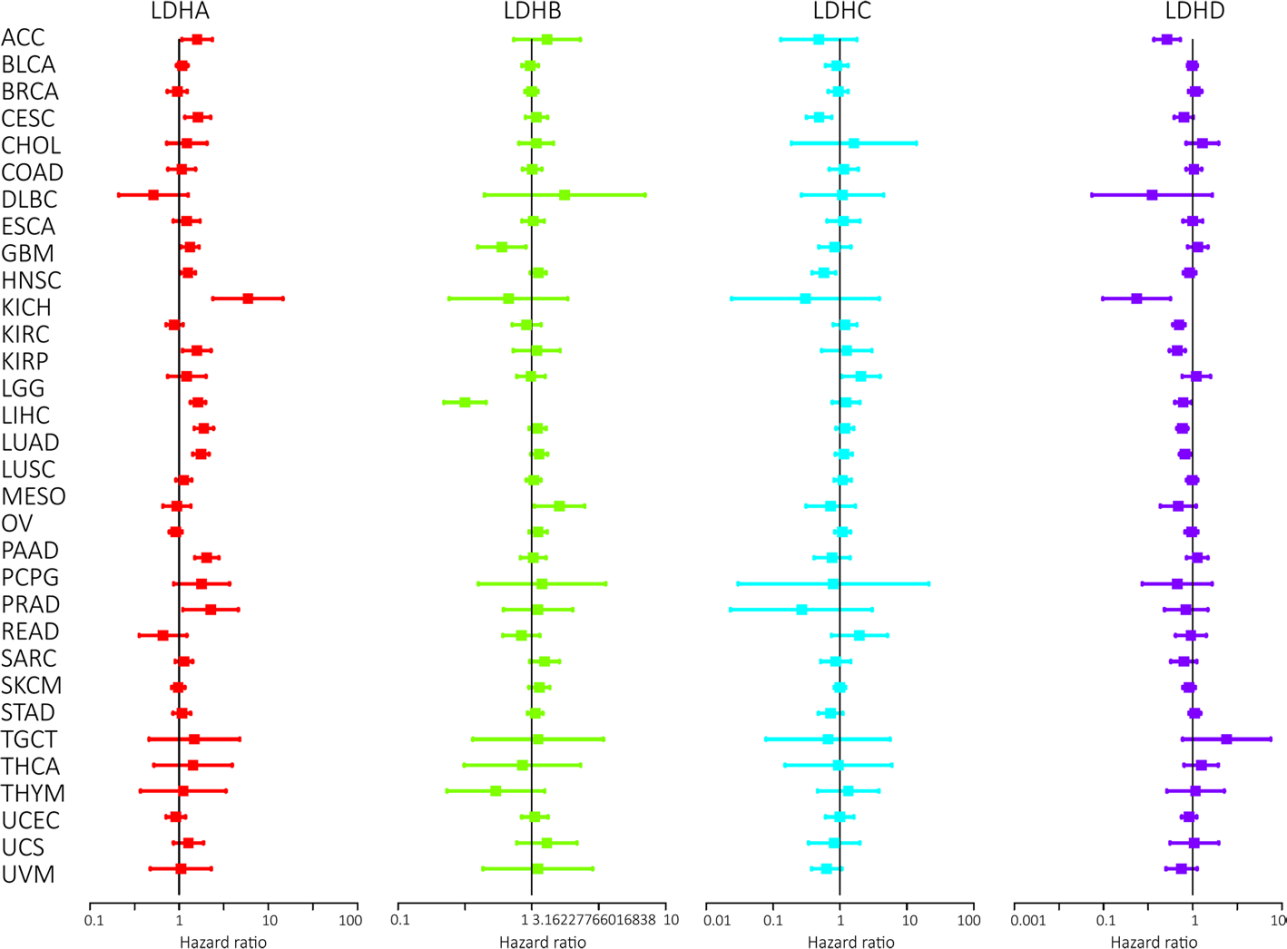
Different colored lines represent the risk values of different genes in the tumor in the cox regression analysis of the association between LDHs family gene expression and survival. A risk ratio < 1 indicates a low risk and a risk ratio > 1 indicates a high risk

The analysis of the Progression Free Interval (PFI) data showed that LDHA expression was significantly associated with ACC (HR = 1.771), GBM (HR = 1.311), HNSC (HR = 1.269), KICH (HR = 3.238), KIRP (HR = 1.522), LGG (HR = 1.545), LIHC (HR = 1.475), LUAD (HR = 1.482), PAAD (HR = 1.865), PCPG (HR = 2.105), PRAD (HR = 1.649) and THYM (HR = 3.061) (Fig. 2C). LDHB expression significantly affected GBM (HR = 0.514), LGG (HR = 0.325), PRAD (HR = 0.820), STAD (HR = 1.167) and THCA (HR = 0.523). LDHC expression was significantly associated with CESC (HR = 0.654), HNSC (HR = 0.653), THYM (HR = 2.123) and UVM (HR = 0.581). LDHD expression significantly affected ACC (HR = 0.736), KICH (HR = 0.466), KIRC (HR = 0.624), KIRP (HR = 0.662), LGG (HR = 0.764), LIHC (HR = 0.835), LUAD (HR = 0.837) and UVM (HR = 0.676). We then evaluated these four genes prognostic value in different tumors. To confirm the accuracy of these candidate markers, we analyzed the predictive ability of LDHs genes for patient prognosis using ROC curves. Our results suggest that LDHs family genes may have good prognostic value in BRCA, COAD, HNSC, KICH, KIRC, KIRP, LUSC, READ, STAD and THCA (Fig. S3A-J). We also analyzed the prognostic risk of LDHs in pan-cancer using cox regression. The results were consistent with the Kaplan–Meier survival curves. The different colored lines represent the risk values of the different genes present in the tumor. Risk ratios < 1 indicate low risk, while risk ratios > 1 indicate high risk (Fig. 3 and Table S1).

### Analysis of genetic alterations in LDHs in pan-cancer

**Fig. 4:**
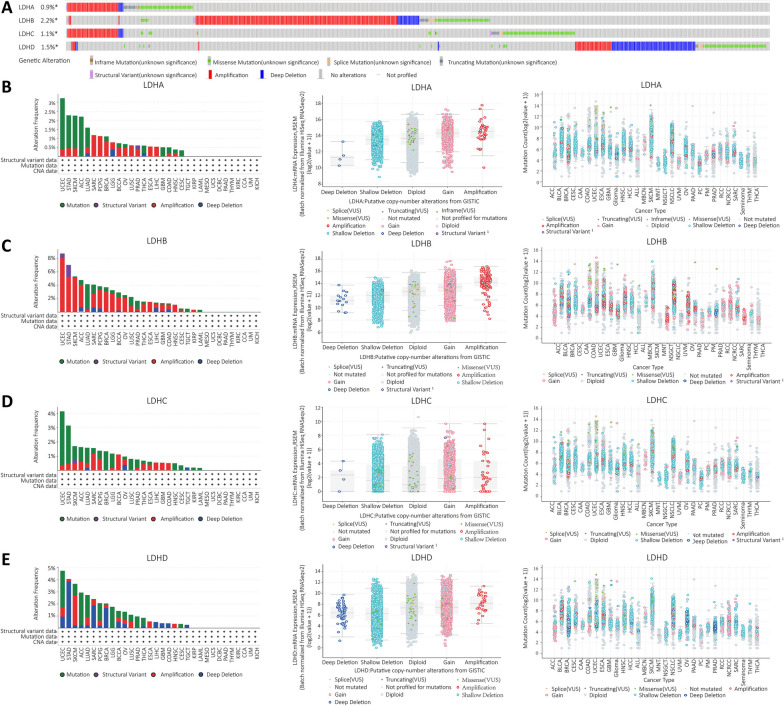
Characterization of the genetic alterations in the LDHs families.** A** General profile of genetic alterations in LDHs families from the pan-cancer dataset in the cBioportal database;** B** Frequency of LDHA alterations from the cBioportal database, dot plots showing the correlation between LDHA copy number and mRNA expression in cBioportal and the number of LDHA mutations in the pan-cancer dataset; **C** Alteration frequency of LDHB from the cBioportal database, dot plots showing the correlation between copy number and mRNA expression of LDHB from cBioportal and the number of LDHB mutations in pan-cancer;** D** From the cBioportal database, the dot plot shows the correlation between copy number and mRNA expression of LDHC from cBioportal and the number of mutations in LDHC in pan-cancer; **E** The frequency of LDHD alterations from the cBioportal database, the dot plot shows the correlation between LDHD copy number and mRNA expression in cBioportal and the number of LDHD mutations in pan-cancer

We analyzed the mutations of LDHs genes in all tumor tissues using the public database of cBioportal. 10,953 patients from the TCGA database were analyzed. The copy number alteration and mutation data of LDHs in pan-cancer are shown in Fig. 4A. LDHB is the most altered of the LDHs, and the main genetic alteration type is amplification and deep deletion. The genetic alteration type of LDHA gene is mutation, which is most common in UCEC, STAD and SKCM. We also investigated the relationship between the different types of LDHA mutations and mRNA expression. The mRNA expression of LDHA with deep detection was lower compared to other types of LDHA alterations. Figure 4B shows the number of LDHA gene mutations in different cancer patients. The type of genetic alteration of the LDHB gene was dominated by amplification. The mRNA expression of the LDHB gene with deep detection was lower compared to the other types of LDHB alterations. mRNA counts of the LDHB gene in different cancer patients are shown in Fig. 4C. The types of genetic alterations of the LDHC gene were dominated by the mutation type. Compared to other types of LDHC alterations, the mRNA expression of LDHC genes with diploid was lower. Figure 4D shows the number of mutations of LDHC genes in patients with different types of cancer. The genetic alteration types of LDHD genes were dominated by mutation. The mRNA expression of LDHD genes with shallow deletion was lower compared to other types of LDHD alterations. The mutation numbers of LDHD genes in different cancer patients are shown in Fig. 4E.

### Correlating LDHs expression with DNA methylation in different cancers

**Fig. 5:**
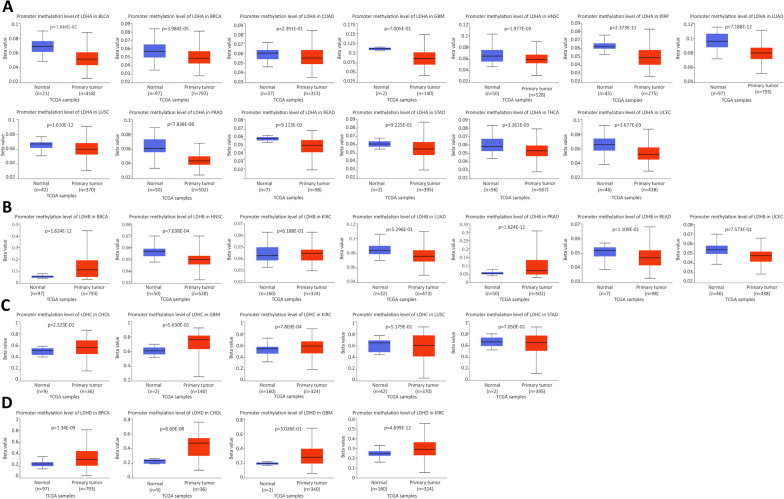
Promoter methylation levels of LDHs in cancers. **A** The promoter methylation levels of LDHA in different types of cancer; **B** The promoter methylation levels of LDHB in different types of cancer;** C** LDHC promoter methylation in different cancers; **D** Promoter methylation levels of LDHD in different cancers

The UALCAN online tool was used to determine promoter methylation levels of genes in different cancer patients and normal populations. β values indicate DNA methylation levels ranging from 0 (unmethylated) to 1 (fully methylated). Hypermethylation (β-value: 0.7–0.5) and hypomethylation (β-value: 0.3–0.25) were considered as different β-critical values. As shown in Fig. 5A, the promoter methylation level of LDHA was significantly lower in 13 tumor groups than in the normal group. The promoter methylation level of LDHB was significantly lower in 4 tumor groups than in the normal group. The promoter methylation level of LDHB was significantly higher than that of the normal group in 3 tumor groups (Fig. 5B). The promoter methylation level of LDHC was significantly lower than that of the normal group in 2 tumor groups, but significantly higher than that of the normal group in 3 tumor groups (Fig. 5C). The promoter methylation level of LDHD was significantly higher in the 4 tumor groups than in the normal group (Fig. 5D). In conclusion, changes in the methylation levels of LDHs promoters were observed in most cancers.

### Biological functions of LDHs in pan-cancer

GSEA was performed to explore the major biological functional processes affected by LDHs in pan-cancer and to screen the signaling pathways positively regulated by LDHs in different cancers. Fig. S4A shows the signaling pathways positively regulated by LDHA in LGG, LIHC and STAD. The signaling pathways positively regulated by LDHB in BRCA, HNSC, LIHC and STAD are shown in Fig. S4B. The signaling pathways positively regulated by LDHC in BRCA, KICH and STAD are shown in Fig. S4C. Fig. S4D reveals the signaling pathways positively regulated by LDHD in HNSC, KICH and LIHC. Analyzing the above data, we found that drug metabolism regulated by LDHs is the most common signaling pathway involved in pan-cancer, followed by JAK-STAT, DNA replication, Toll-like receptor and T cell receptor signaling pathways, and fatty acid metabolism.

### Correlation analysis between LDHs and molecular and immune subtypes in pan-cancers

Using the TCGA and TISIDB databases, we investigated the correlation between LDHs expression and molecular and immune subtypes in different cancer types. The immune subtypes were classified as C1 (wound healing), C2 (IFN-γ dominant), C3 (inflammatory), C4 (lymphocyte depleted), C5 (immunologically quiet) and C6 (TGF-β dominant). We analyzed the differences in the expression of the LDHs gene between the immune subtypes in the different types of cancer using the TCGA database. The results showed that LDHs expression was significantly correlated with different immune subtypes in BLCA、BRCA、KIRC、LGG、LIHC、LUAD、OV、PRAD and UCEC patients (Fig. S5A-J). Using the TISIDB database, the correlation of LDHA expression with molecular subtypes in different cancers was performed. The results showed that LDHA expression was correlated with BRCA (*p* = 1.1e-22), HNSC (*p* = 2.78e-20), KIRP (*p* = 2.24e-05), LGG (*p* = 7.69e-42), PCPG (*p* = 6.46e-12), PRAD (*p* = 1.07e-05), STAD (*p* = 1.29e-11) and UCEC (*p* = 3. 21e-11) (Fig. S6A). LDHB expression was significantly correlated with the different molecular isoforms of BRCA (*p* = 3.17e-75), ESCA (*p* = 2.17e-06), HNSC (*p* = 9.13e-08), LGG (*p* = 3.23e-24), OV (*p* = 2.6e-07), PCPG (*p* = 3.35e-06) and PRAD (*p* = 5.43e-08) (Fig. S6B). LDHC expression was significantly correlated with BRCA (*p* = 8.22e-10), HNSC (*p* = 9.5e-06), OV (*p* = 2.87e-03), PCPG (*p* = 3.97e-03), STAD (*p* = 9.1e-05) and UCEC (*p* = 7.84e-07) (Fig. S6C). LDHD expression was significantly correlated with different molecular isoforms of BRCA (*p* = 2.48e-45), COAD (*p* = 3.79e-05), ESCA (*p* = 1.28e-04), HNSC (*p* = 2.26e-05), LGG (*p* = 8.87e-11), LIHC (*p* = 3.86e-08), PRAD (*p* = 7.13e-20), STAD (*p* = 4.82e-06) and UCEC (*p* = 1.92e-09) (Fig. S6D). In conclusion, LDHs expression may have an impact on the molecular and immune subtypes of different cancer types.

### Correlation of LDHs expression with immune-related biomarkers

**Fig. 6:**
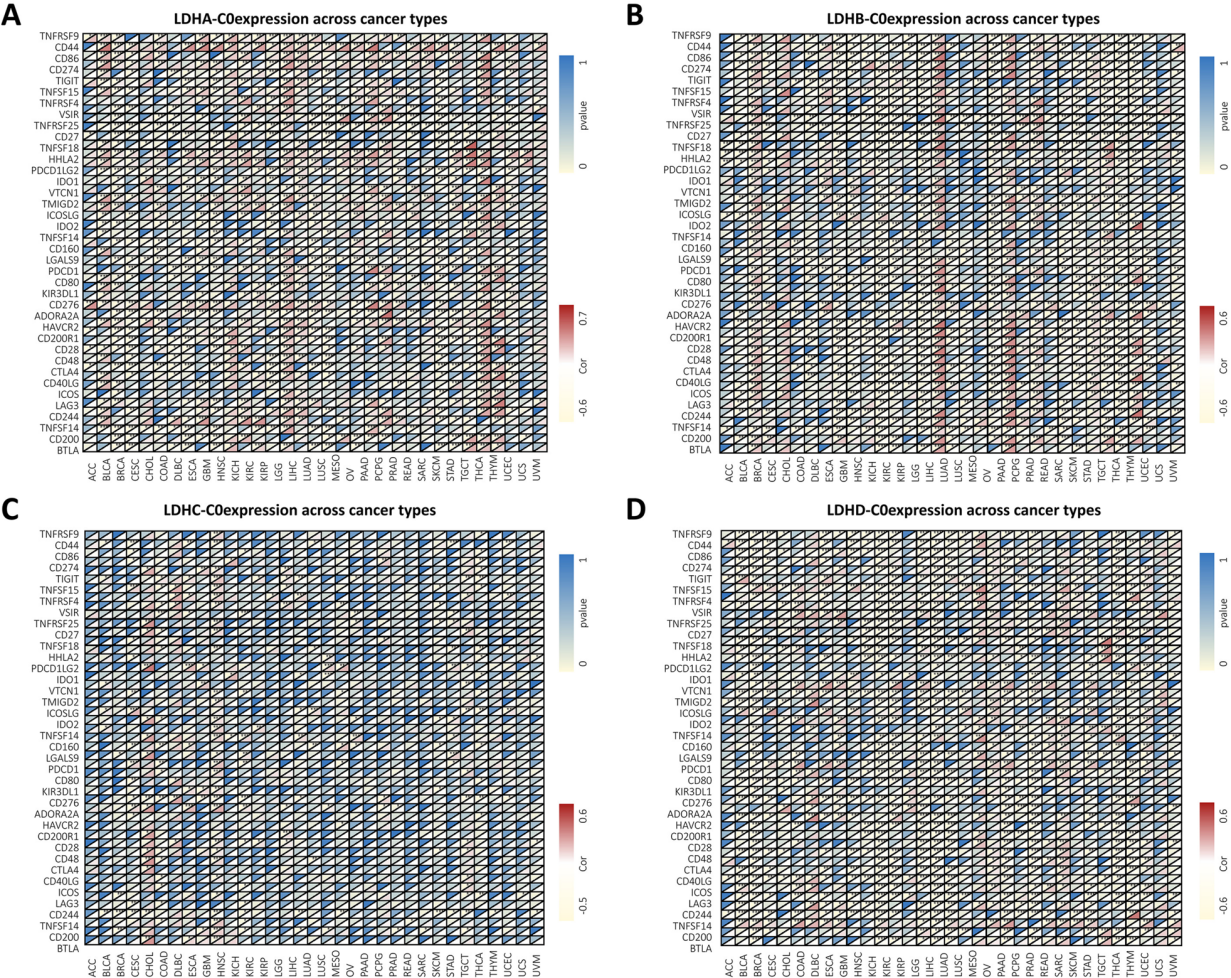
Relationship between the expression of LDHs in pan-cancer and genes of immune checkpoint (ICP). Co-expression relationship between immune checkpoint (ICP) genes and LDHA (**A**), LDHB (**B**), LDHC(**C**) and LDHD (**D**)

Correlation between LDHs gene expression and immune checkpoints was analyzed to investigate the effect of LDHs gene on immunity in pan-cancer. As shown in Fig. 6A-D, the immune checkpoint (ICP) gene has a strong impact on immune cell infiltration and immunotherapy. The association between LDHs expression and ICP genes in human cancers was then examined. A close association was found between 47 ICP genes in many cancers. This suggests that LDHs may coordinate the activity of these ICP genes in different cancers and may serve as ideal immunotherapeutic targets.

### Association between LDHs and TME and stemness scores in pan-cancers

**Fig. 7:**
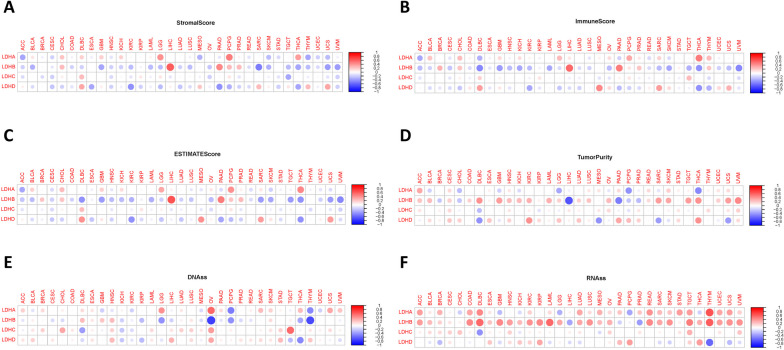
Correlation between the expression of LDHs family genes and the tumor microenvironment and stemness score in pan-cancer. **A-D** Correlation between LDHs family gene expression in pan-cancer and StromalScore, ImmuneScore, ESTIMATESocre and TumorPurity scores. Red dots indicate a positive correlation between expression in the tumor and StromalScore. Blue dots indicate a negative correlation between expression in the tumor and StromalScore; **E–F** Correlation between the expression of genes of the LDHs family in pan-cancer and DNAss and RNAss. Red dots indicate a positive correlation between oncogene expression and immune score, blue dots indicate a negative correlation

We investigated the relationship between LDHs expression and TME in pan-cancer. and found that LDHs can affect TME and stemness scores in a variety of cancers and visualize the results (Fig. 7A-F).

### Correlating LDHs expression with TMB, MSI and immune modulators

**Fig. 8:**
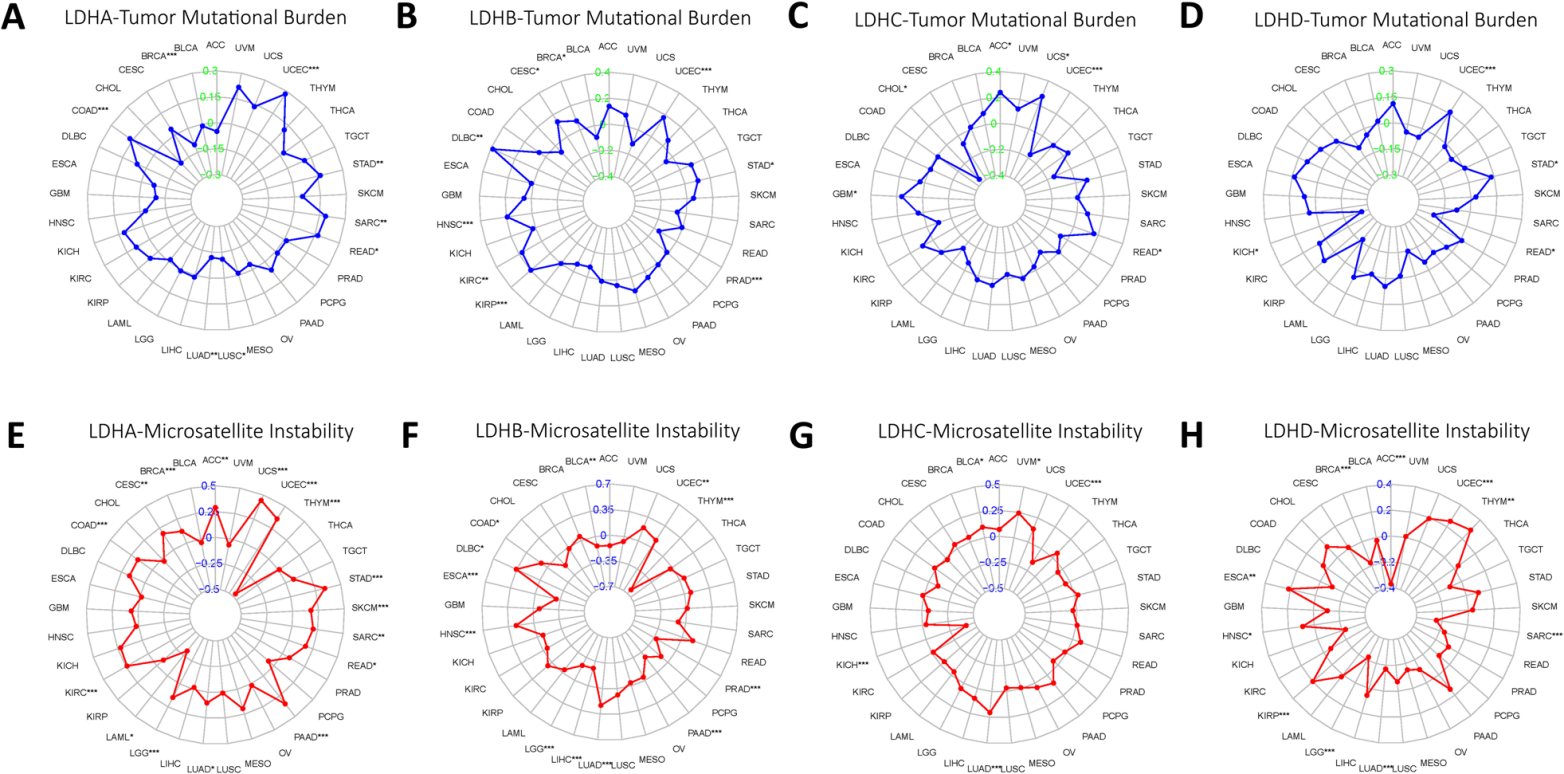
Correlation of TMB and MSI with LDHs family gene expression. **A**-**D** Correlation between TMB and LDHs family gene expression; **E**–**H** Correlation between MSI and LDHs family gene expression

The correlation between TMB and MSI in the LDHs of different types of cancer was analyzed. The results showed that LDHA expression was significantly positively correlated with TMB in UCEC, STAD, SARC, READ and COAD, but negatively correlated with TMB in LUSC, LUAD and BRCA. LDHB expression was significantly positively correlated with TMB in UCEC, STAD, KIRP, KIRC, HNSC, DLBC, CESC and BRCA. LDHB expression appeared to be negatively correlated with TMB in PRAD. In ACC, UCS, READ and GBM, the expression of LDHC was positively correlated with TMB, in UCEC and CHOL, the expression of LDHC was significantly negatively correlated with TMB. In UCEC and STAD, the expression of LDHD was positively correlated with TMB, and in READ and KICH, the expression of LDHD was negatively correlated with TMB (Fig. 8A-D). As shown in Fig. 8E-H, LDHA expression was significantly positively correlated with MSI in ACC、UCS、UCEC、STAD、SKCM、SARC、READ、PAAD、LUAD、LGG、KIRC、COAD、CESC and BRCA, but was significantly negatively correlated with MSI in THYM and LAML. The expression of LDHB was significantly positively correlated with MSI in UCEC、LUAD、HNSC、DLBC and COAD, but negatively correlated with MSI in THYM、PRAD、PAAD、LIHC、LGG、ESCA and BLCA. LDHC expression was positively correlated with MSI in UVM, LUAD and BLCA. In UCEC and KICH, LDHC expression was significantly negatively correlated with MSI. In UCEC、THYM、KIRP、HNSC and ESCA, the expression of LDHD was positively correlated with MSI, while in AAC、SARC、LUAD、LGG and BRCA it was negatively correlated with MSI. Using the TISIDB database, we further investigated the relationship between the three immunomodulators and LDHs expression. It was found that there was a correlation between the expression of LDHs and immunomodulators (immunostimulator, immunoinhibitor and MHC molecules) (Fig. S7A-D). Taken together, the above results further support the speculation of LDHs may have an effect on anti-tumor immunity by modulating immune mechanisms in certain cancer patients.

### Analysis of LDHs subtype pharmacoresponse

**Fig. 9:**
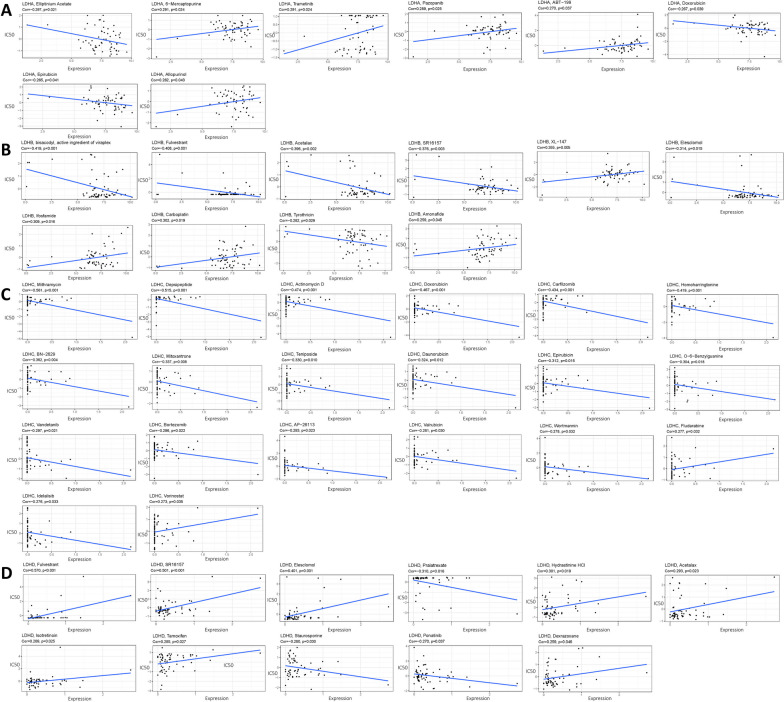
Correlation between LDHs family genes and Pearson's drug sensitivity scores in different tumor cell lines from the CellMiner database. drug sensitivity analysis of LDHs family genes. Drug sensitivity was analyzed with LDHA (**A**), LDHB (**B**), LDHC (**C**) and LDHD (**D**), the X-axis indicates the relative sensitivity to certain drugs, the Y-axis indicates the relative expression of LDHs

We used the CellMiner database to further explore the analysis of potential correlations between pharmacoresensitivity and LDHs. In particular, LDHA expression was negatively correlated with elliptinium acetate and doxorubicum, but positively correlated with 6-mercaptopurine and trametinib. LDHB expression was negatively correlated with the bisacodyl component of Viraplex. The expression of LDHC was negatively correlated with mithramycin and depsipeptide, but positively correlated with fludarabine and vorinostat. LDHD expression was negatively correlated with fulvestrant and SR16157, but positively correlated with fulvestrant (Fig. 9A-D).

### Association between LDHD gene expression and clinicopathological features as well as immune cell infiltration in HCC

This study systematically analyzed the role of LDHs subtype in pan-cancer through gene expression, prognosis, methylation, mutation patterns, immune infiltration and functional enrichment analysis. LDHD was found to play a critical role in prognosticating HCC patients based on clinical practice and immune infiltration. To further investigate the correlation between LDHD gene expression and clinicopathological features of HCC, we obtained HCC data from the TCGA database, including transcriptomic data and clinicopathological data. Wilcoxon signed-rank test was used to analyze the difference in the expression of LDHD mRNA in HCC tissues and normal tissues. The results showed that the expression of LDHD gene in HCC tissues was significantly lower than that in normal tissues (*p* = 1.823e-10) (Fig. S8A), suggesting that LDHD may be an oncogene in the malignant development of HCC. To investigate whether the LDHD gene may act as an oncogene to influence the prognosis of HCC patients, we analyzed the relationship between the expression level of LDHD in HCC and the overall survival (OS) and progression-free survival (PFS) of patients. The results showed that the OS and PFS of patients in the low LDHD gene expression group was shorter than that of patients in the high LDHD gene expression group (*P* = 0.037, *P* = 0.024, respectively) (Fig. S8B-C). The results suggest that LDHD may be affect the prognosis of HCC patients. HCC patients were divided into high expression group and low expression group according to LDHD gene median expression levels. Age, gender, race, pathological stage, histological stage, T stage and AFP were found to be statistically different between the two groups, suggesting that differences in LDHD gene expression levels may affect the clinicopathological progression and prognostic survival of patients (Table S2). We also analyzed the relationship between LDHD gene expression and clinicopathological variables in HCC patients using Wilcoxon signed rank test and logistic regression. The results showed that LDHD gene expression in HCC was significantly associated with age (*p* = 1.484e-05) (Fig. S8D), gender (*p* = 0.021) (Fig. S8E), T stage (*p* = 3.254e-04) (Fig. S8F), histological grade (*p* = 8.281e-07) (Fig. S8G), pathological stage (*p* = 6.252e-04) (Fig. S8H) and AFP (*p* = 9.817e-13) (Fig. S8I). Univariate logistic regression was used to analyze the association between LDHD gene expression and clinicopathological characteristics of HCC patients. The results showed that LDHD gene expression was significantly associated with age, gender, race, T stage, histological grade, pathological stage, tumor status and AFP (Table S3). These results suggest that LDHD gene has the potential to be an indicator of HCC stage.

Univariate and multivariate Cox regression analyses are commonly used to find factors associated with patient prognosis. Univariate Cox regression showed that pathological stage, T stage and LDHD gene expression were risk factors for HCC. Multifactorial Cox regression analysis showed that LDHD gene expression was an independent prognostic factor associated with overall survival in patients with HCC (HR, 0.74; CI, 0.631–0.869; *P* = 0.000) (Table S4).

In addition, immune cell infiltration also has a significant impact on the prognosis of HCC patients. Analysis of the relationship between LDHD gene expression and immune cell infiltration found that LDHD expression was significantly correlated with macrophage (*p* = 0.03), B-cell memory (*p* = 0.028) and T-cell follicular assist (*p* = 0.02) (Fig. S8J). This suggests that LDHD gene may affect the prognosis of HCC patients by influencing immune cell infiltration. The above results suggest that LDHD may have an impact on the clinicopathological features and immune cell infiltration of HCC patients, thereby affecting their survival and prognosis.

### LDHD knockdown or overexpression affects the proliferation of HCC cells.

**Fig. 10:**
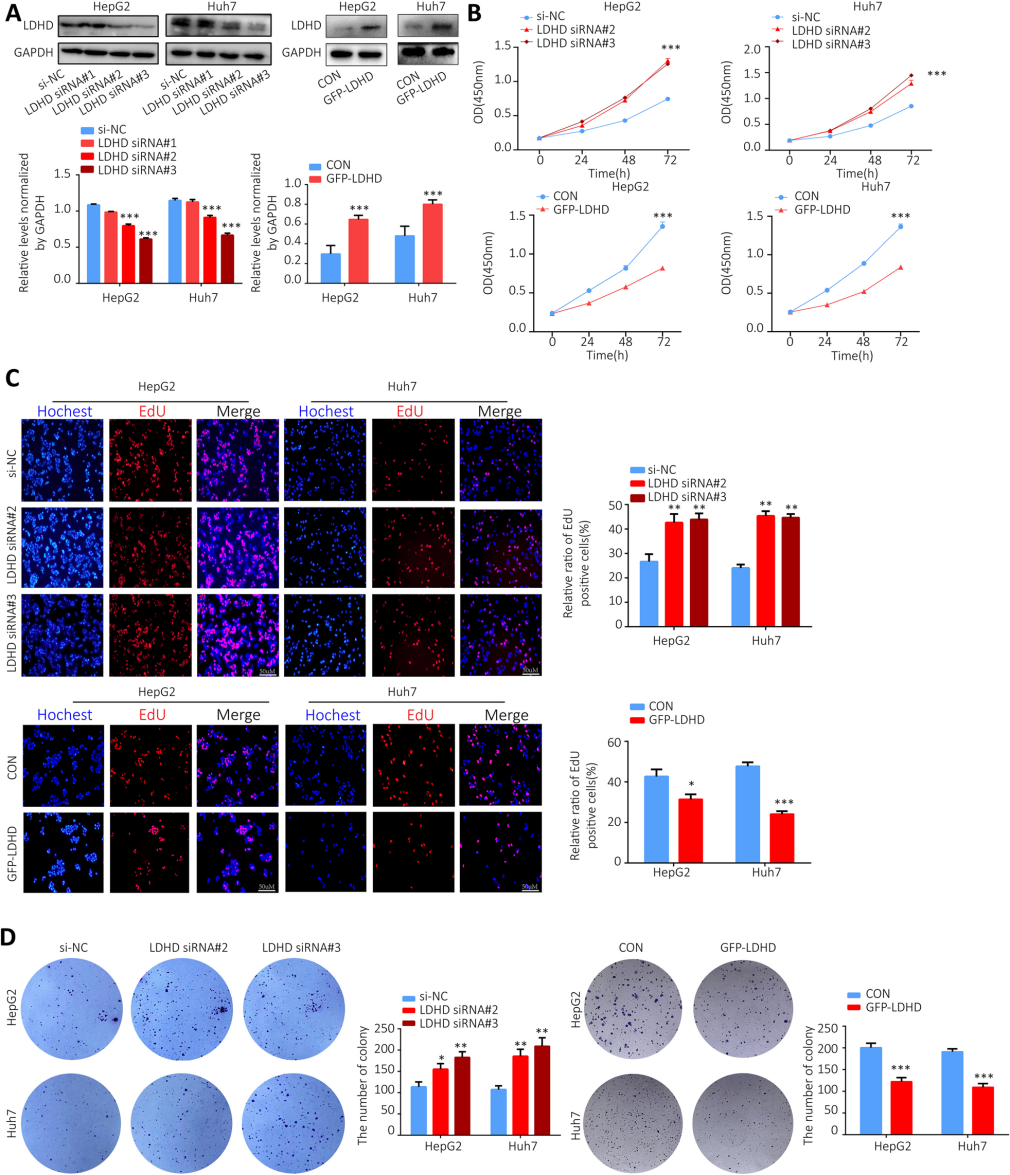
Knockdown or overexpression of LDHD affects the proliferation of HCC cell. **A** Western Blotting was used to detect the transfection efficiency of LDHD siRNA and GFP-LDHD in HepG2 and Huh7 cells; **B** CCK-8 assay was used to detect the cell viability; The effects of LDHD siRNA and GFP-LDHD on the proliferation of HepG2 and Huh7 cells were detected using EdU (**C**) and colony formation (**D**) assays. *P < 0.05, **P < 0.01, ***P < 0.001 vs. NC group

To further investigate the effect of LDHD on HCC proliferation, we down-regulated and up-regulated the expression level of LDHD in HepG2 and Huh7 cell lines by LDHD siRNA and GFP-tagged LDHD overexpression plasmid (GFP-LDHD) transfection, and verified the transfection efficiency using western blotting (Fig. 10A). Due to their obvious interference efficiency, LDHD siRNA#2 and LDHD siRNA#3 were selected to subsequent experiments. CCK-8, EdU and colony formation assays showed that knockdown of LDHD significantly increased HepG2 and Huh7 cell viability, promoted cell proliferation. Moreover, LDHD overexpression showed the opposite effects (Fig. 10B-D).

### Knockdown or overexpression of LDHD affects HCC cell migration and invasion

**Fig. 11:**
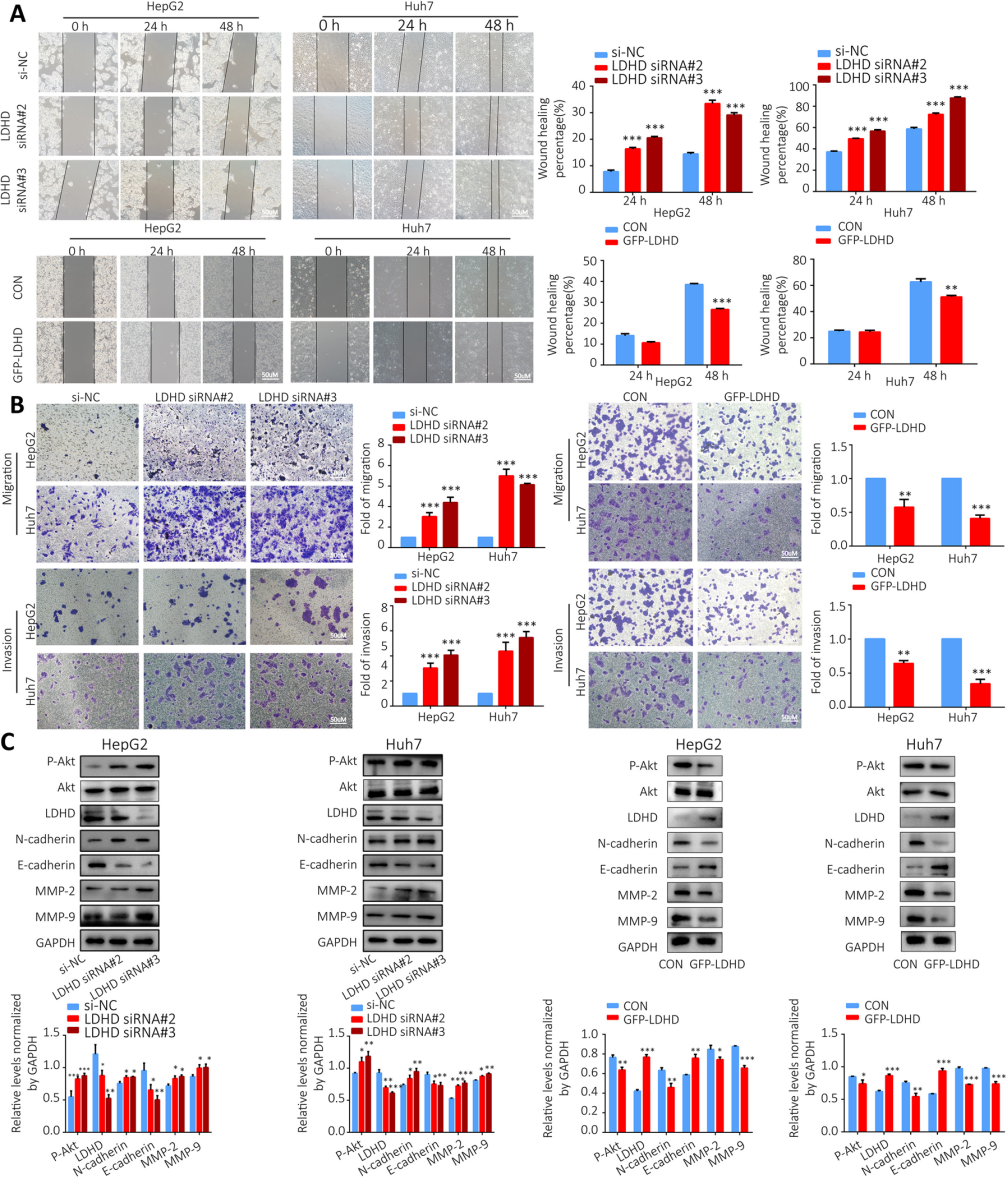
Knockdown or overexpression of LDHD affects HCC cell migration and invasion.** A** Wound healing assay was used to investigate the effects of LDHD siRNA and GFP-LDHD on HepG2 and Huh7 cell migration; **B** Transwell assay was used to determine the effects of LDHD siRNA and GFP-LDHD on HepG2 and Huh7 cell migration and invasion; **C** Western Blotting was performed to measure the expression levels of N-cadherin, E-cadherin, MMP-2, MMP-9 and Akt phosphorylation after LDHD siRNA and GFP-LDHD transfection in HepG2 and Huh7 cell lines. *P < 0.05, **P < 0.01, ***P < 0.001 vs. NC group

To investigate the effect of LDHD on the migration and invasion of HepG2 and Huh7 cells. Wound healing assay and transwell assay were performed following transfection of LDHD siRNA or GFP-LDHD plasmids. Figure 11A-B showed that LDHD knockdown significantly promoted HepG2 and Huh7 cell migration and invasion compared to the negative control group. However, LDHD overexpression significantly inhibited the cells migration and invasion. The expression levels of MMP-2, MMP-9 and N-cadherin showed a clear upregulation after transfection with LDHD siRNA, while E-cadherin expression showed a significant decrease. After transfection of GFP-LDHD, the expression levels of MMP-2, MMP-9, N-cadherin and E-cadherin all showed the opposite changes to LDHD knockdown. In addition, compared with negative control transfected cells, the phosphorylation level of Akt in HepG2 and Huh7 cells transfected with LDHD siRNA was significantly increased, but significantly decreased in GFP-LDHD transfected cells (Fig. 11C).

## Discussion

Cancer cells undergo a metabolic shift towards anaerobic glycolysis, and LDHs play a crucial role in this process [31]. Increased expression of LDHs has been observed in many patients with advanced cancer, making them a potential diagnostic marker for cancer. Analyzing the expression and prognosis of LDHs in cancer revealed that LDHs were differentially expressed in specific cancers and were correlated with the prognostic survival of patients. These suggest that LDHs subtype may impact the biological progression of certain cancers. Preliminary interesting experimental results prompted us to further analyze LDHs subtype in pan-cancer from the aspects of methylation, mutation patterns, immune infiltration, functional enrichment analysis and potential chemotherapeutic agents.

DNA methylation regulates gene expression at an epigenetic level. Aberrant DNA methylation is associated with malignancy. Gene expression levels generally show an opposite change to the DNA methylation level [32]. Our result showed that LDHs subtype can influence DNA methylation levels in certain cancers. This suggests that LDHs subtype may impact cancer progression by affecting DNA methylation. Analysis of LDHs subtype mutations in pan-cancers using the cBioportal database revealed that LDHs was mutated in most tumors. These results suggest an association between mutations in the LDHs gene and pan-cancer. Correlation analyses also indicate an association between LDHs expression and molecular and immune subtypes of different cancer types.

The immune microenvironment in tumor tissues translates into tumor heterogeneity and affects the clinical efficacy of anticancer drugs. Therefore, the correlation between LDHs expression and StromalScore, ImmuneScore, ESTIMAEScore and TumorPurity in pan-cancer were analyzed. We further investigated the correlation of LDHs expression with stemness score DNAss and RNAss, which revealed the association of LDHs with specific cancer types (BRCA, LGG, LIHC, and LUAD). As immune checkpoint therapy for tumors becomes increasing effective, the relationship between immune checkpoint-related genes in patients with different tumor types of LDHs was analyzed. The results showed that LDHs significantly affect the expression of immune checkpoint-related genes in various tumor types. This suggests that LDHs may serve as a potential class of therapeutic targets and play an important role in tumor immunity and tumor microenvironment. The findings provide a new direction for future combinational targeted immunotherapy. In recent years, TMB and MSI have emerged as predictive markers for the efficacy of tumor immunotherapy, TMB reflects the extent of cancer mutations, and tumors with high TMB tend to have higher levels of neoantigens presented to T cells via major histocompatibility complex (MHC) proteins. This help the immune system to recognize tumors and activate the anti-tumor effects of stem cells [33]. Therefore, higher TMB levels usually indicate better immunotherapeutic efficacy. MSI is a phenotype resulting from defects in DNA mismatch repair. It is associated with increased cancer susceptibility and is considered an important biomarker for immune checkpoint blockade therapy [34]. In this study, we analyzed the correlation between LDHs and TMB as well as MSI. The data showed that the expression of LDHs correlates with TMB and MSI in a variety of cancer types. The result suggests that LDHs may be novel biomarkers for predicting a patient’s response to immunotherapy.

Enrichment analysis using GSEA indicated a significant association between LDHs subtype and classic cancer-related pathways. Drug sensitivity analysis conducted on the CellMiner database showed that LDHs subtype was associated with anticancer drugs. These findings suggest that the expression of LDHs subtype can predict the therapeutic effect of drugs and influence the response to targeted molecular therapeutics, making LDHs a potential new target for future cancer therapy.

Upon analyzing the expression, clinical practice and immune infiltration of LDHs subtype in various cancers, it was discovered that LDHD plays a key role in the prognosis and immune infiltration of HCC patients. Analysis of the differential expression of LDHs subtype across cancer patients revealed that LDHD expression was low in HCC patients. Furthermore, LDHD expression was found to be highly correlated with patient prognosis and clinicopathological features in HCC patients. To further analyze the clinical significance of LDHD differential expression in HCC patients, logistic regression analysis revealed that the LDHD gene has the potential to be an indicator of HCC stage, Multivariate Cox regression analysis showed that LDHD gene expression was an independent prognostic factor associated with OS in HCC patients. Therefore, it can be concluded that the expression of the LDHD gene is important for the treatment of patients with HCC. We also found that LDHD expression was associated with molecular subtypes and immunological subtypes of HCC patients, suggesting that LDHD may influence the prognosis of HCC patients through immune cell infiltration.

To verify the reliability of the results, the experiments were performed at the cellular level. It has been reported that MMP-2 and MMP-9 are the major enzymes responsible for degrading type IV collagen and they play an important role in the metastasis and invasion of HCC [35]. Epithelial-mesenchymal transition (EMT) is a complex biotransformation process that enables epithelial cells to temporarily acquire mesenchymal characteristics. EMT is considered to be an important factor in cancer invasion and metastasis [36]. Akt/PKB (protein kinase B), a serine-threonine kinase, is involved in a variety of cellular pathways, including survival, proliferation, invasion, apoptosis and angiogenesis [37]. We investigated the effects of LDHD on the biological behavior in vitro, and the underlying molecular mechanism. Our data showed that LDHD significantly affected the viability, proliferation and migration ability of HepG2 and Huh7 cells. Additionally, it affected the expression of MMP-2, MMP-9, N-cadherin, E-cadherin and Akt phosphorylation. These findings suggest that LDHD inhibits the proliferation and migration of HCC cells through affecting the Akt signaling pathway, MMPs expression, and EMT.

## Conclusion

Our study was limited to online databases retrieval rather than physical data collection. To enhance the reliability of the results cross-validation methods were employed with multiple databases. Although our results are primarily based on bioinformatics analysis and require experimental validation, this study can provide new research directions. In addition, both bioinformatics analyses and in vitro experiments suggest that LDHD may be a potential biomolecular marker and immunotherapeutic target in HCC.

### Supplementary Information

**Additional file 1:** **Fig. S1.** Correlation between expression of LDHs family genes and overall survival of patients with different TCGA cancer types. A. Survival curve analysis between LHDA gene expression and overall survival of patients with ACC, CESC, LGG, LIHC, LUAD and PAAD; B. Survival curve analysis between LDHB gene expression and overall survival in patients with GBM, HNSC, LGG, LIHC, LUAD and SKCM; C. Survival curve analysis between LDHC gene expression and overall survival of UCEC and UVM patients; D. Survival curve analysis between LDHD gene expression and overall survival of patients with ACC, CESC, KIRC, KIRP, LUAD and UVM. **Fig.** **S2.** Protein expression levels of LDHA family genes in different cancer tissues and normal tissues based on the HPA database. IHC images of LDHA (A), LDHB (B), LDHC (C) and LDHD (D) in lung and lung cancer, thyroid and thyroid cancer, kidney and kidney cancer from the HPA database. **Fig. S3.** Assessment of the prognostic value of the LDHs family genes in pan-cancer patients. ROC curve analysis of LDHs family genes to assess the utility of LDHs as a prognostic marker in BRCA patients (A), COAD patients (B); HNSC patients (C); KICH patients (D), KIRC patients (E), KIRP patients (F), LUSC patients (G); READ patients (H), STAD patients(I) and THCA patients (J). **Fig. S4.** Biological functions of LDHs in pan-cancer. A. KEGG signature of LDHA in LGG, LIHC and STAD for GSEA analysis; B. GSEA analysis of KEGG features of LDHB in BRCA, HNSC, LIHC and STAD; C. GSEA analysis of KEGG features of LDHC in BRCA, KICH and STAD; D. GSEA analysis of KEGG features of LDHD in HNSC, KICH and LIHC. **Fig. S5.** Correlation of LDHs expression in pan-cancer and immune subtypes. Correlation of LDHs expression with immune subtypes in BLCA (A), BRCA (B), KIRC (C), KIRP (D), LGG (E), LIHC (F), LUAD (G), OV (H), PRAD (I), and UCEC (J). **Fig. S6.** Association between LDHs family gene expression and pan-cancer molecular subtypes. A. Correlation between LDHA expression and BRCA, HNSC, KIRP, LGG, PCPG, PRAD, STAD and UCEC molecular subtypes; B. Correlation between LDHB expression and BRCA, ESCA, HNSC, LGG, OV, PCPG and PRAD molecular subtypes; C. Correlation between LDHC expression and BRCA, HNSC, OV, PCPG, STAD and UCEC molecular subtypes; D. Correlation between LDHD expression and BRCA, COAD, ESCA, HNSC, LGG, LIHC, PRAD, STAD and UCEC molecular subtypes. **Fig. S7.** Correlation between the expression of genes of the LDHs family in pan-cancer and immune-related molecules based on the analysis of the TISIDB database. Correlation between the expression of LDHA (A), LDHB (B), LDHC (C) and LDHD (D) in pan-cancer and immune inhibitors, immune stimulators and MHC molecules in the TISIDB database. **Fig. S8.** Association between LDHD gene expression and clinicopathological features as well as immune cell infiltration in HCC. A. LDHD gene expression in HCC tissues and normal tissues; B-C. Relationship between LDHD expression levels and OS, PFS in HCC patients. Association between LDHD gene expression in HCC and age (D), gender (E), T stage (F), Histological grade (G), Pathological stage (H) and AFP (I). (J) Association between LDHD gene expression and immune cell infiltration.

**Additional file 2:** **Table S1.** Prognostic risk of LDH in pan-cancer analysed by Cox regression. Different coloured lines represent risk values for different genes in the tumour. Risk ratios <1 indicate low risk and risk ratios >1 indicate high risk.

**Additional file 3:** **Table S2.** TCGA liver cancer patient characteristics.

**Additional file 4:** **Table S3.** LDHD expression correlated with clinical pathological characteristics (logistic regression).

**Additional file 5:** **Table S4.** Univariate analysis and multivariate analyses of liver cancer patient overall survival.

## Data Availability

The data are available in the TCGA (https://portal.gdc.cancer.gov/) database and can be downloaded directly.

## References

[1] Siegel RL, Miller KD, Wagle NS, Jemal A (2023). Cancer statistics, 2023. CA.

[2] Sung H, Ferlay J, Siegel RL, Laversanne M, Soerjomataram I, Jemal A (2021). Global cancer statistics 2020: GLOBOCAN estimates of incidence and mortality worldwide for 36 cancers in 185 Countries. CA.

[3] Onkar SS, Carleton NM, Lucas PC, Bruno TC, Lee AV, Vignali DAA (2023). The great immune escape: understanding the divergent immune response in breast cancer subtypes. Cancer Discov.

[4] Wang Q, Zhang X, Wei W, Cao M (2022). PET imaging of lung cancers in precision medicine: current landscape and future perspective. Mol Pharm.

[5] Piawah S, Venook AP (2019). Targeted therapy for colorectal cancer metastases: a review of current methods of molecularly targeted therapy and the use of tumor biomarkers in the treatment of metastatic colorectal cancer. Cancer.

[6] Shimasaki N, Jain A, Campana D (2020). NK cells for cancer immunotherapy. Nat Rev Drug Discovery.

[7] Kwon MJ (2022). Predictive biomarkers for molecularly targeted therapies and immunotherapies in breast cancer. Arch Pharmacal Res.

[8] Sun L, Zhang H, Gao P (2022). Metabolic reprogramming and epigenetic modifications on the path to cancer. Protein Cell.

[9] Feng J, Li J, Wu L, Yu Q, Ji J, Wu J (2020). Emerging roles and the regulation of aerobic glycolysis in hepatocellular carcinoma. J Exper Clin Cancer Res.

[10] Ždralević M, Marchiq I, de Padua MMC, Parks SK, Pouysségur J (2017). Metabolic plasiticy in cancers-distinct role of glycolytic enzymes gpi, ldhs or membrane transporters MCTs. Front Oncol.

[11] Mathupala SP, Ko YH, Pedersen PL (2009). Hexokinase-2 bound to mitochondria: cancer's stygian link to the "Warburg Effect" and a pivotal target for effective therapy. Semin Cancer Biol.

[12] Li S, Li J, Dai W, Zhang Q, Feng J, Wu L (2017). Genistein suppresses aerobic glycolysis and induces hepatocellular carcinoma cell death. Br J Cancer.

[13] Claps G, Faouzi S, Quidville V, Chehade F, Shen S, Vagner S (2022). The multiple roles of LDH in cancer. Nat Rev Clin Oncol.

[14] Urbańska K, Orzechowski A (2019). Unappreciated and to control apoptosis and autophagy in tumor cells. Int J Mol Sci.

[15] Sheng SL, Liu JJ, Dai YH, Sun XG, Xiong XP, Huang G (2012). Knockdown of lactate dehydrogenase A suppresses tumor growth and metastasis of human hepatocellular carcinoma. FEBS J.

[16] Yuan JQ, Wang SM, Guo L (2023). S100A9 promotes glycolytic activity in HER2-positive breast cancer to induce immunosuppression in the tumour microenvironment. Heliyon.

[17] Ding Z, Yang J, Wu B, Wu Y, Guo F (2023). Long non-coding RNA CCHE1 modulates LDHA-mediated glycolysis and confers chemoresistance to melanoma cells. Cancer Metab.

[18] Khajah MA, Khushaish S, Luqmani YA (2021). Lactate Dehydrogenase A or B knockdown reduces lactate production and inhibits breast cancer cell motility in vitro. Front Pharmacol.

[19] Cui J, Quan M, Jiang W, Hu H, Jiao F, Li N (2015). Suppressed expression of LDHB promotes pancreatic cancer progression via inducing glycolytic phenotype. Med Oncol (Northwood, London, England).

[20] Wang JM, Jiang JY, Zhang DL, Du X, Wu T, Du ZX (2021). HYOU1 facilitates proliferation, invasion and glycolysis of papillary thyroid cancer via stabilizing LDHB mRNA. J Cell Mol Med.

[21] Chen L, Wu Q, Xu X, Yang C, You J, Chen F (2021). Cancer/testis antigen LDHC promotes proliferation and metastasis by activating the PI3K/Akt/GSK-3β-signaling pathway and the in lung adenocarcinoma. Exp Cell Res.

[22] Kong L, Du W, Cui Z, Wang L, Yang Z, Zhang H (2016). Expression of lactate dehydrogenase C in MDA-MB-231 cells and its role in tumor invasion and migration. Mol Med Rep.

[23] Wang Y, Li G, Wan F, Dai B, Ye D (2018). Prognostic value of D-lactate dehydrogenase in patients with clear cell renal cell carcinoma. Oncol Lett.

[24] Song KJ, Yu XN, Lv T, Chen YL, Diao YC, Liu SL (2018). Expression and prognostic value of lactate dehydrogenase-A and -D subunits in human uterine myoma and uterine sarcoma. Medicine.

[25] Papanicolau-Sengos A, Aldape K (2022). DNA methylation profiling: an emerging paradigm for cancer diagnosis. Annu Rev Pathol.

[26] Samstein RM, Lee CH, Shoushtari AN, Hellmann MD, Shen R, Janjigian YY (2019). Tumor mutational load predicts survival after immunotherapy across multiple cancer types. Nat Genet.

[27] Taieb J, Svrcek M, Cohen R, Basile D, Tougeron D, Phelip JM (2022). Deficient mismatch repair/microsatellite unstable colorectal cancer: Diagnosis, prognosis and treatment. European J Cancer.

[28] Bader JE, Voss K, Rathmell JC (2020). Targeting metabolism to improve the tumor microenvironment for cancer immunotherapy. Mol Cell.

[29] Zhang Y, Zhang Z (2020). The history and advances in cancer immunotherapy: understanding the characteristics of tumor-infiltrating immune cells and their therapeutic implications. Cell Mol Immunol.

[30] Sisodiya SM (2021). Precision medicine and therapies of the future. Epilepsia.

[31] Miholjcic TBS, Halse H, Bonvalet M, Bigorgne A, Rouanne M, Dercle L (2023). Rationale for LDH-targeted cancer immunotherapy. European J Cancer (Oxford, England : 1990).

[32] Huang W, Li H, Yu Q, Xiao W, Wang DO (2022). LncRNA-mediated DNA methylation: an emerging mechanism in cancer and beyond. J Exper Clin Cancer Res.

[33] Jiao D, Zhang J, Chen P, Guo X, Qiao J, Zhu J (2021). HN1L promotes migration and invasion of breast cancer by up-regulating the expression of HMGB1. J Cell Mol Med.

[34] Baretti M, Le DT (2018). DNA mismatch repair in cancer. Pharmacol Ther.

[35] Espíndola KMM, Ferreira RG, Narvaez LEM, Silva Rosario ACR, da Silva AHM, Silva AGB (2019). Chemical and pharmacological aspects of caffeic acid and its activity in hepatocarcinoma. Front Oncol.

[36] Scheau C, Badarau IA, Costache R, Caruntu C, Mihai GL, Didilescu AC (2019). The role of matrix metalloproteinases in the epithelial-mesenchymal transition of hepatocellular carcinoma. Anal Cell Pathol (Amst).

[37] Manning BD, Toker A (2017). AKT/PKB signaling: navigating the network. Cell.

