# Supplementary material for: Systematic analysis of the role of LDHs subtype in pan-cancer demonstrates the importance of LDHD in the prognosis of hepatocellular carcinoma patients
Source: BMC Cancer. 2024 Jan 31;24:156. doi: 10.1186/s12885-024-11920-8 (PMC10829303; doi:10.1186/s12885-024-11920-8)
Supplement: Supplementary file 1 — Additional file 1: Fig. S1. Correlation between expression of LDHs family genes and overall survival of patients with different TCGA cancer types. A. Survival curve analysis between LHDA gene expression and overall survival of patients with ACC, CESC, LGG, LIHC, LUAD and PAAD; B. Survival curve analysis between LDHB gene expression and overall survival in patients with GBM, HNSC, LGG, LIHC, LUAD and SKCM; C. Survival curve analysis between LDHC gene expression and overall survival of UCEC and UVM patients; D. Survival curve analysis between LDHD gene expression and overall survival of patients with ACC, CESC, KIRC, KIRP, LUAD and UVM. Fig. S2. Protein expression levels of LDHA family genes in different cancer tissues and normal tissues based on the HPA database. IHC images of LDHA (A), LDHB (B), LDHC (C) and LDHD (D) in lung and lung cancer, thyroid and thyroid cancer, kidney and kidney cancer from the HPA database. Fig. S3. Assessment of the prognostic value of the LDHs family genes in pan-cancer patients. ROC curve analysis of LDHs family genes to assess the utility of LDHs as a prognostic marker in BRCA patients (A), COAD patients (B); HNSC patients (C); KICH patients (D), KIRC patients (E), KIRP patients (F), LUSC patients (G); READ patients (H), STAD patients(I) and THCA patients (J). Fig. S4. Biological functions of LDHs in pan-cancer. A. KEGG signature of LDHA in LGG, LIHC and STAD for GSEA analysis; B. GSEA analysis of KEGG features of LDHB in BRCA, HNSC, LIHC and STAD; C. GSEA analysis of KEGG features of LDHC in BRCA, KICH and STAD; D. GSEA analysis of KEGG features of LDHD in HNSC, KICH and LIHC. Fig. S5. Correlation of LDHs expression in pan-cancer and immune subtypes. Correlation of LDHs expression with immune subtypes in BLCA (A), BRCA (B), KIRC (C), KIRP (D), LGG (E), LIHC (F), LUAD (G), OV (H), PRAD (I), and UCEC (J). Fig. S6. Association between LDHs family gene expression and pan-cancer molecular subtypes. A. Correlation between LDHA expression and [file 12885_2024_11920_MOESM1_ESM.docx]

Systematic analysis of the role of LDHs subtype in pan-cancer demonstrates the importance of LDHD in the prognosis of hepatocellular carcinoma patients

Shengnan Wang^1,2,3+^, Xingwei Wu^1,2,4,+^, Xiaoming Wu^1,2,5^, Jin Cheng^1,2,6^, Qianyi Chen^1,2^，Zhilin Qi^1,2*^

^1^Department of Biochemistry and Molecular Biology, Wannan Medical College, Wuhu, Anhui 241002, P.R. China;

^2^Anhui Province Key Laboratory of Active Biological Macro-molecules, Wannan Medical College, Wuhu, Anhui 241002, P.R. China;

^3^Department of Pathology, Fuyang People's Hospital, Anhui Medical University, Fuyang, Anhui, 236000, P.R. China;

^4^Clinical Laboratory, Traditional Chinese Hospital of Lu'an, Anhui University of Chinese Medicine, Lu'an 237000, Anhui, P.R. China;

^5^Department of Thyroid and Breast Surgery, Yijishan Hospital, First Affiliated Hospital of Wannan Medical College, Wuhu, Anhui, 241002, P.R. China;

^6^Department of Gastroenterology, Yijishan Hospital, First Affiliated Hospital of Wannan Medical College, Wuhu, Anhui, 241002, P.R. China;

+ These authors have contributed equally to this work.

* Corresponding authors

Zhilin Qi, E-mail: [20010012@wnmc.edu.cn](mailto:20010012@wnmc.edu.cn). Department of Biochemistry and Molecular biology, Wannan Medical College, No.22 Wenchang West Road, Wuhu, Anhui 241002, P.R. China.

**Supplement figures and tables**


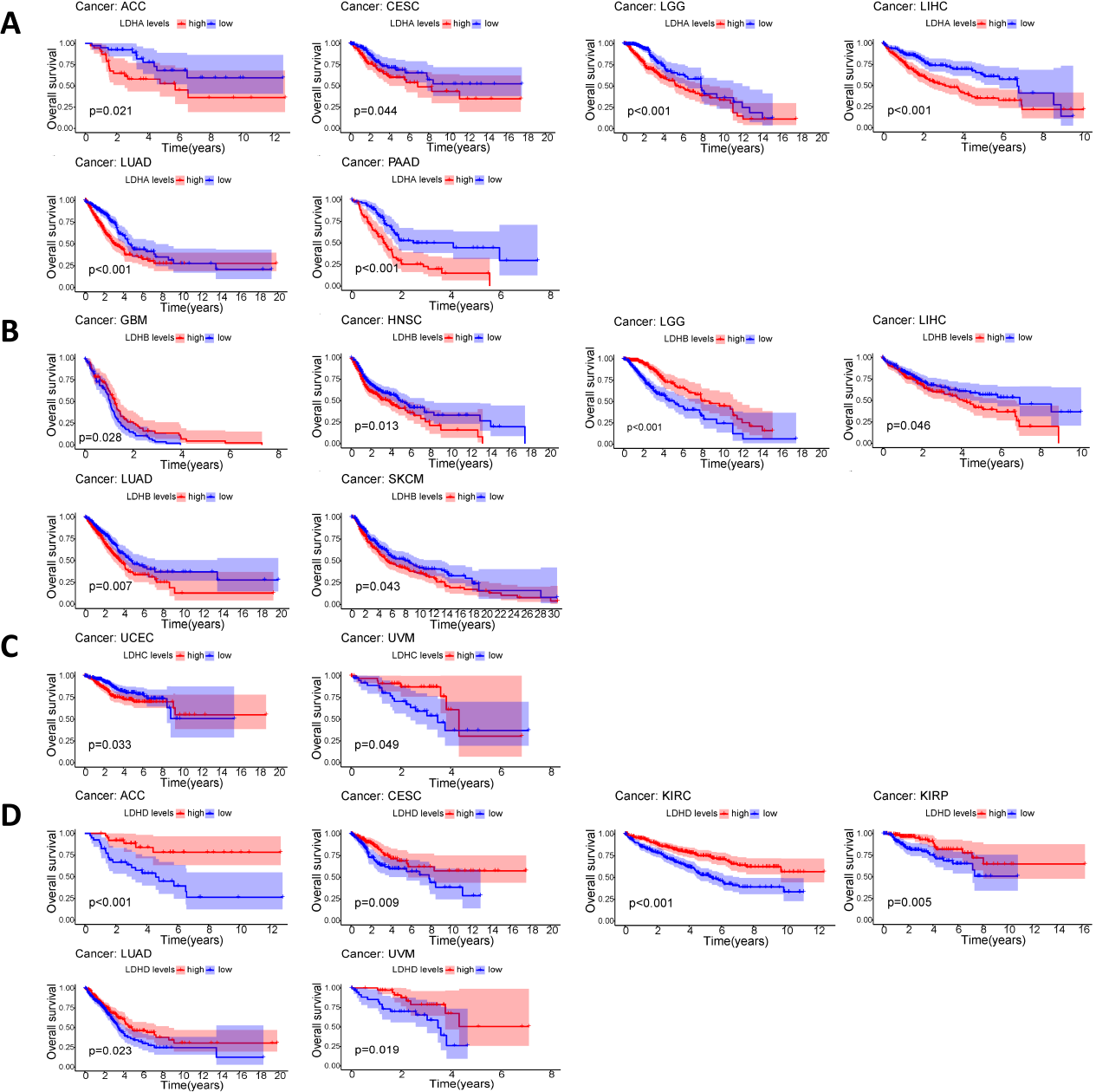


**Fig. S1** Correlation between expression of LDHs family genes and overall survival of patients with different TCGA cancer types. **A.** Survival curve analysis between LHDA gene expression and overall survival of patients with ACC, CESC, LGG, LIHC, LUAD and PAAD; **B.** Survival curve analysis between LDHB gene expression and overall survival in patients with GBM, HNSC, LGG, LIHC, LUAD and SKCM; **C.** Survival curve analysis between LDHC gene expression and overall survival of UCEC and UVM patients; **D.** Survival curve analysis between LDHD gene expression and overall survival of patients with ACC, CESC, KIRC, KIRP, LUAD and UVM.


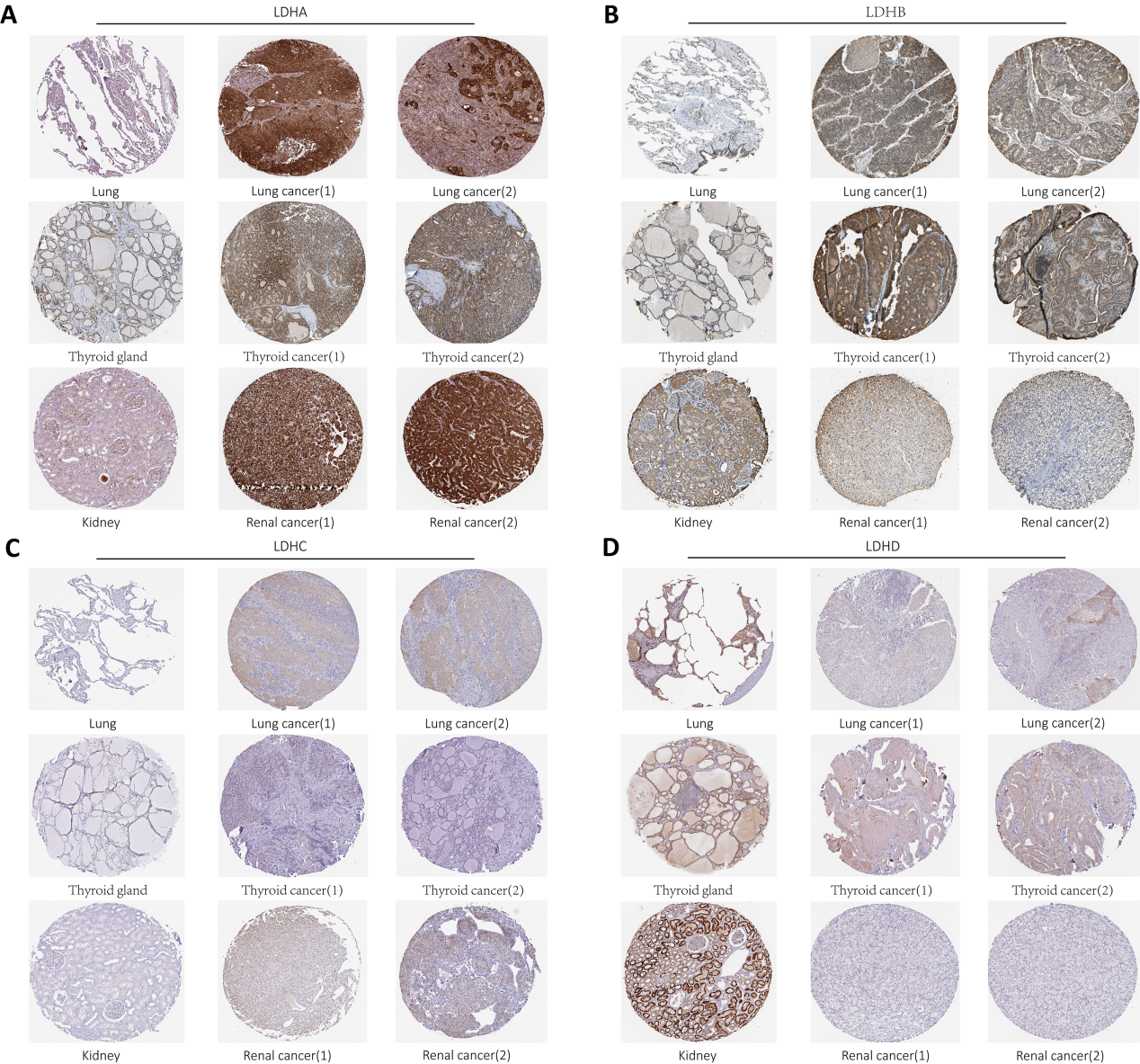


**Fig. S2** Protein expression levels of LDHA family genes in different cancer tissues and normal tissues based on the HPA database. IHC images of LDHA **(A),** LDHB **(B),** LDHC **(C)** and LDHD **(D)** in lung and lung cancer, thyroid and thyroid cancer, kidney and kidney cancer from the HPA database.


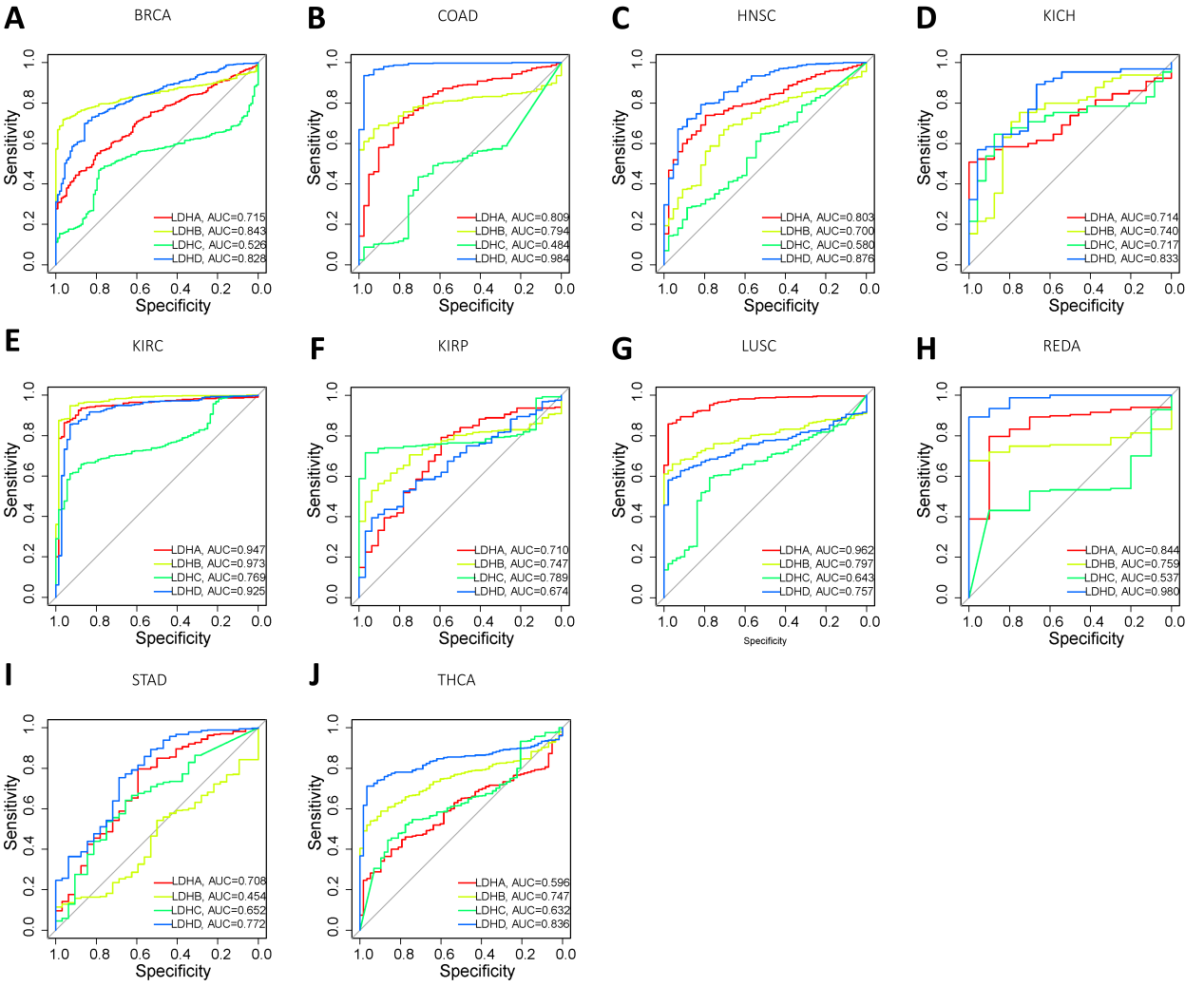


**Fig. S3** Assessment of the prognostic value of the LDHs family genes in pan-cancer patients. ROC curve analysis of LDHs family genes to assess the utility of LDHs as a prognostic marker in BRCA patients **(A),** COAD patients **(B)**; HNSC patients **(C)**; KICH patients **(D)**, KIRC patients **(E)**, KIRP patients **(F)**, LUSC patients **(G)**; READ patients **(H),** STAD patients**(I)** and THCA patients **(J)**.


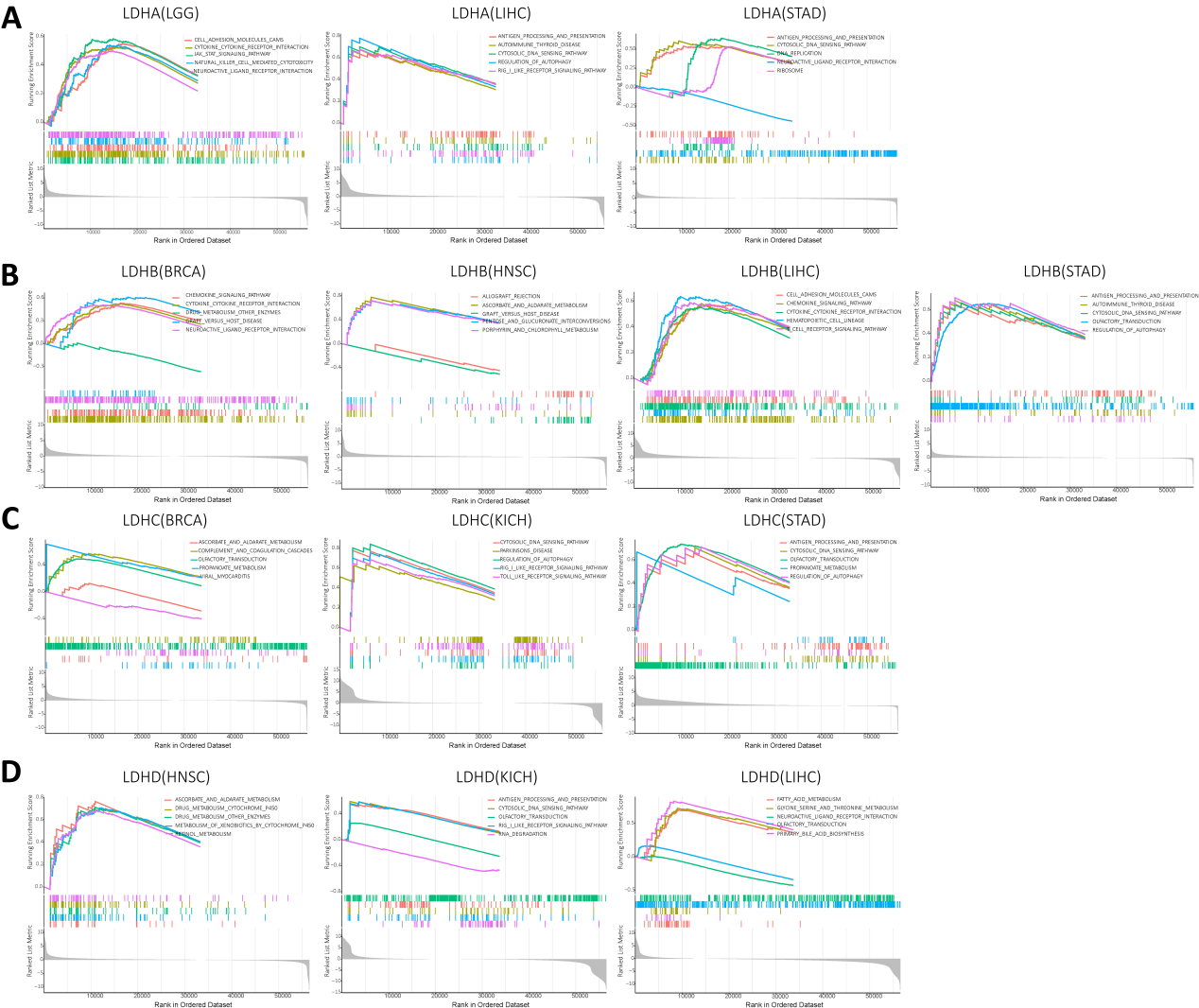


**Fig. S4** Biological functions of LDHs in pan-cancer. **A.** KEGG signature of LDHA in LGG, LIHC and STAD for GSEA analysis; **B.** GSEA analysis of KEGG features of LDHB in BRCA, HNSC, LIHC and STAD; **C.** GSEA analysis of KEGG features of LDHC in BRCA, KICH and STAD; **D.** GSEA analysis of KEGG features of LDHD in HNSC, KICH and LIHC.


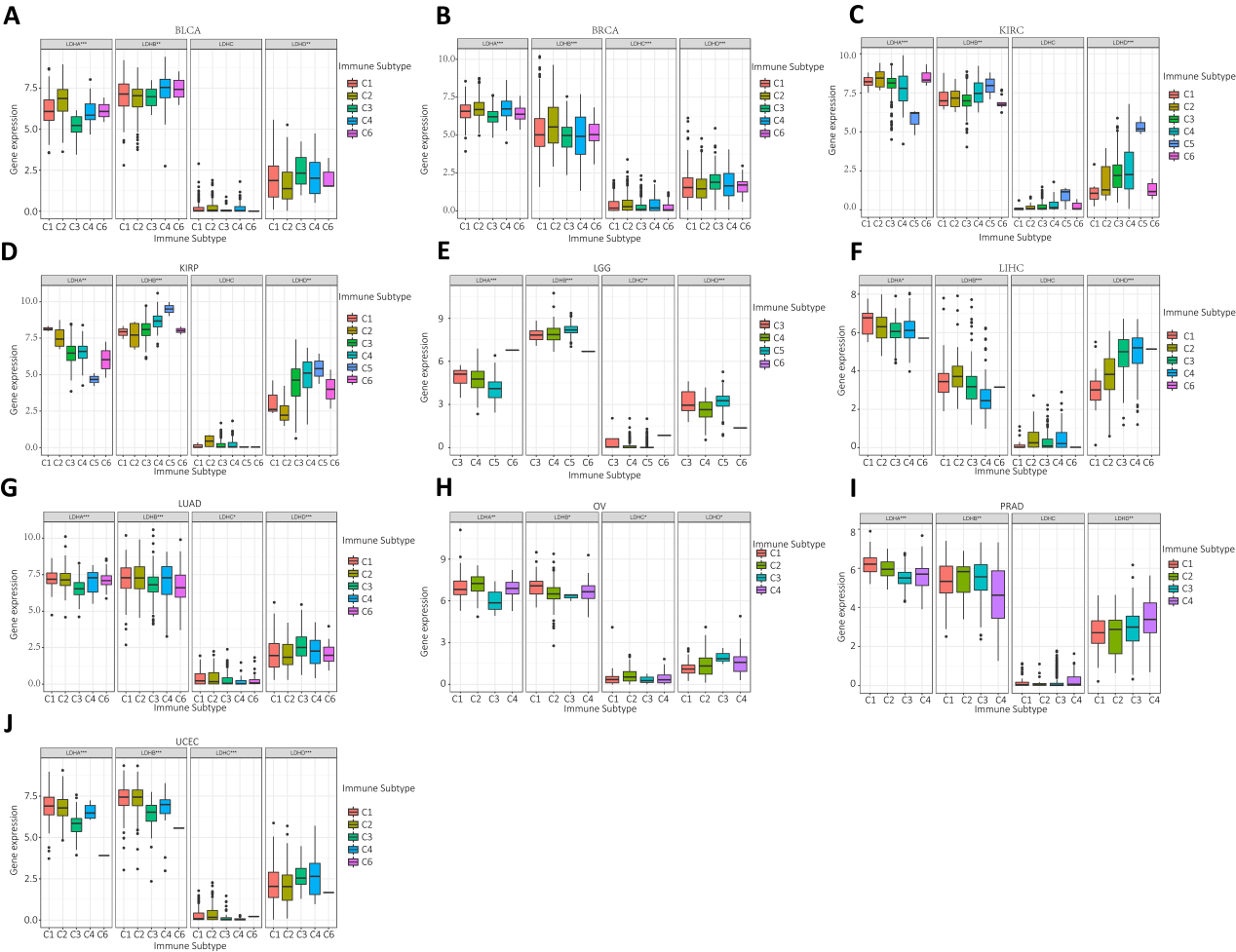


**Fig. S5** Correlation of LDHs expression in pan-cancer and immune subtypes. Correlation of LDHs expression with immune subtypes in BLCA **(A),** BRCA **(B)**, KIRC **(C)**, KIRP **(D)**, LGG **(E)**, LIHC **(F)**, LUAD **(G)**, OV **(H)**, PRAD **(I)**, and UCEC **(J)**.


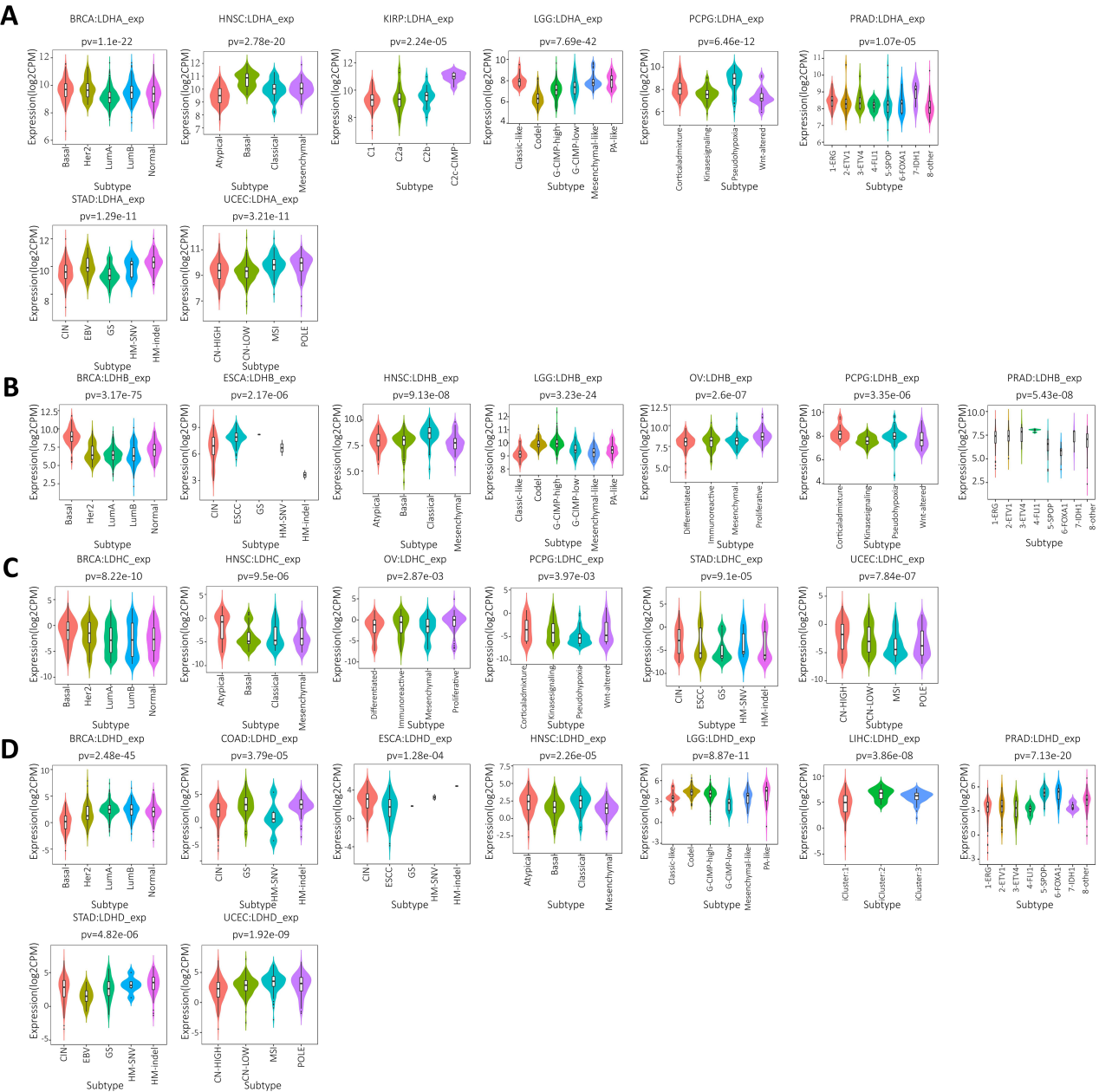


**Fig. S6** Association between LDHs family gene expression and pan-cancer molecular subtypes. **A.** Correlation between LDHA expression and BRCA, HNSC, KIRP, LGG, PCPG, PRAD, STAD and UCEC molecular subtypes; **B.** Correlation between LDHB expression and BRCA, ESCA, HNSC, LGG, OV, PCPG and PRAD molecular subtypes; **C.** Correlation between LDHC expression and BRCA, HNSC, OV, PCPG, STAD and UCEC molecular subtypes; **D.** Correlation between LDHD expression and BRCA, COAD, ESCA, HNSC, LGG, LIHC, PRAD, STAD and UCEC molecular subtypes.


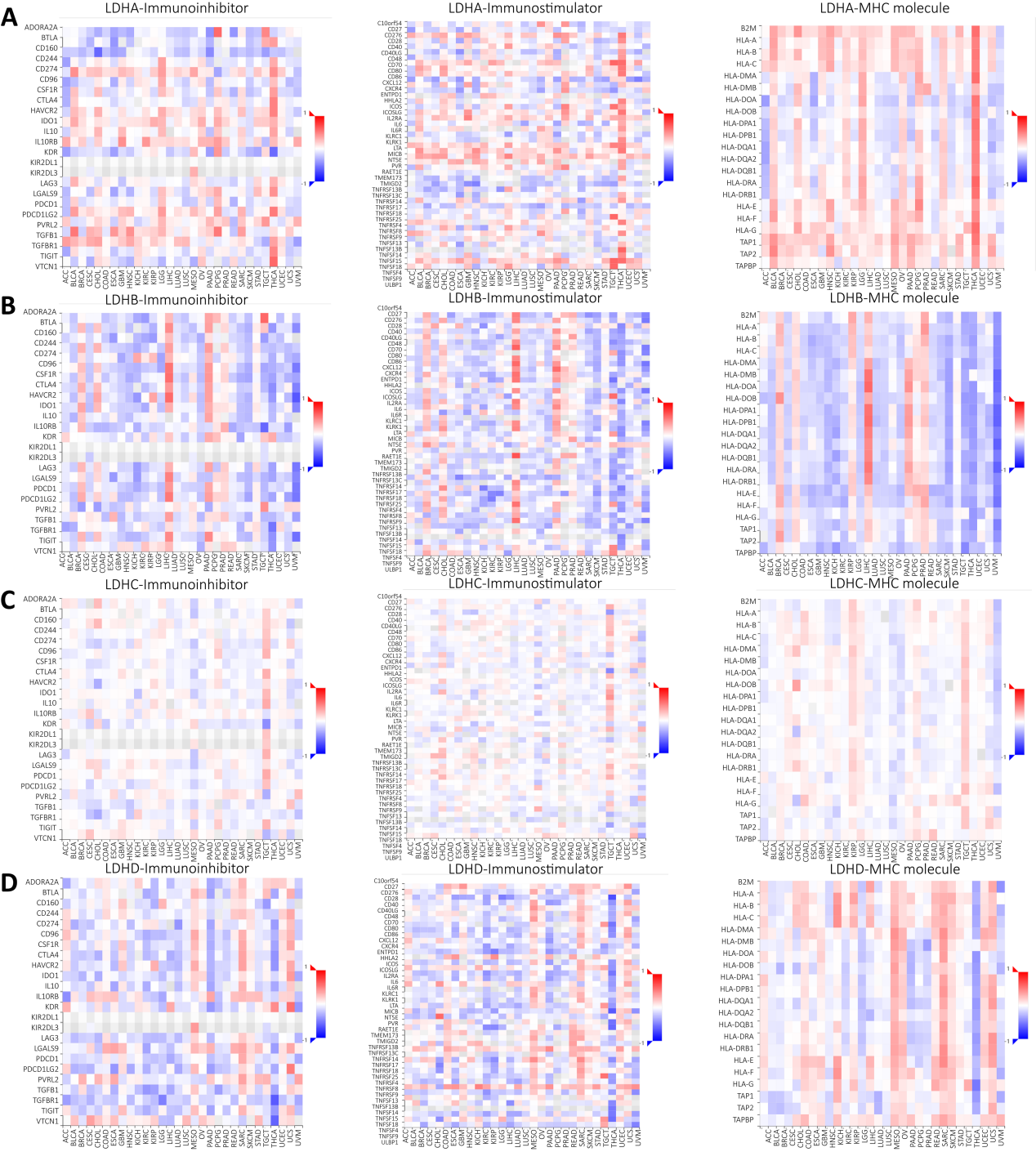


**Fig. S7** Correlation between the expression of genes of the LDHs family in pan-cancer and immune-related molecules based on the analysis of the TISIDB database. Correlation between the expression of LDHA **(A),** LDHB **(B),** LDHC **(C)** and LDHD **(D)** in pan-cancer and immune inhibitors, immune stimulators and MHC molecules in the TISIDB database.


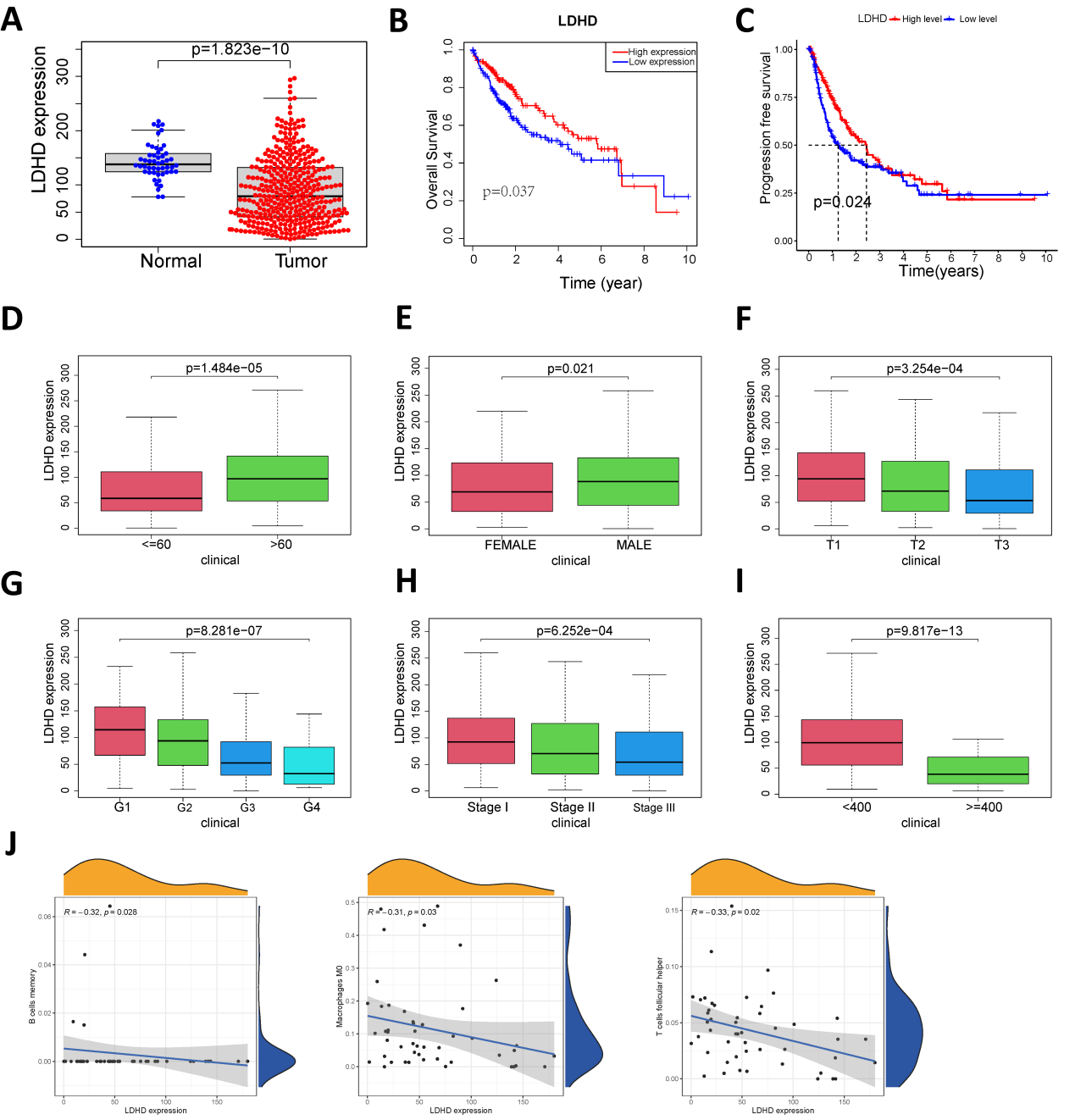


**Fig. S8** Association between LDHD gene expression and clinicopathological features as well as immune cell infiltration in HCC. **A.** LDHD gene expression in HCC tissues and normal tissues; **B-C.** Relationship between LDHD expression levels and OS, PFS in HCC patients. Association between LDHD gene expression in HCC and age **(D)**, gender **(E)**, T stage **(F)**, Histological grade **(G)**, Pathological stage **(H)** and AFP **(I)**. **(J)** Association between LDHD gene expression and immune cell infiltration.
